# Calredoxin regulates the chloroplast NADPH-dependent thioredoxin reductase in *Chlamydomonas reinhardtii*

**DOI:** 10.1093/plphys/kiad426

**Published:** 2023-07-20

**Authors:** Karen Zinzius, Giulia Maria Marchetti, Ronja Fischer, Yuval Milrad, Anne Oltmanns, Simon Kelterborn, Iftach Yacoby, Peter Hegemann, Martin Scholz, Michael Hippler

**Affiliations:** Institute of Plant Biology and Biotechnology, University of Münster, 48143 Münster, Germany; Institute of Plant Biology and Biotechnology, University of Münster, 48143 Münster, Germany; Institute of Plant Biology and Biotechnology, University of Münster, 48143 Münster, Germany; School of Plant Sciences and Food Security, The George S. Wise Faculty of Life Sciences, Tel Aviv University, Tel Aviv 6997801, Israel; Institute of Plant Biology and Biotechnology, University of Münster, 48143 Münster, Germany; Institute of Biology, Experimental Biophysics, Humboldt University of Berlin, 10099 Berlin, Germany; School of Plant Sciences and Food Security, The George S. Wise Faculty of Life Sciences, Tel Aviv University, Tel Aviv 6997801, Israel; Institute of Biology, Experimental Biophysics, Humboldt University of Berlin, 10099 Berlin, Germany; Institute of Plant Biology and Biotechnology, University of Münster, 48143 Münster, Germany; Institute of Plant Biology and Biotechnology, University of Münster, 48143 Münster, Germany; Institute of Plant Science and Resources, Okayama University, Kurashiki, Okayama 710-0046, Japan

## Abstract

Calredoxin (CRX) is a calcium (Ca^2+^)-dependent thioredoxin (TRX) in the chloroplast of Chlamydomonas (*Chlamydomonas reinhardtii*) with a largely unclear physiological role. We elucidated the CRX functionality by performing in-depth quantitative proteomics of wild-type cells compared with a *crx* insertional mutant (IM*_crx_*), two CRISPR/Cas9 KO mutants, and CRX rescues. These analyses revealed that the chloroplast NADPH-dependent TRX reductase (NTRC) is co-regulated with CRX. Electron transfer measurements revealed that CRX inhibits NADPH-dependent reduction of oxidized chloroplast 2-Cys peroxiredoxin (PRX1) via NTRC and that the function of the NADPH-NTRC complex is under strict control of CRX. Via non-reducing SDS-PAGE assays and mass spectrometry, our data also demonstrated that PRX1 is more oxidized under high light (HL) conditions in the absence of CRX. The redox tuning of PRX1 and control of the NADPH-NTRC complex via CRX interconnect redox control with active photosynthetic electron transport and metabolism, as well as Ca^2+^ signaling. In this way, an economic use of NADPH for PRX1 reduction is ensured. The finding that the absence of CRX under HL conditions severely inhibited light-driven CO_2_ fixation underpins the importance of CRX for redox tuning, as well as for efficient photosynthesis.

## Teaser

Calredoxin-dependent redox regulation ensures efficient photosynthesis.

## Introduction

The process of oxygenic photosynthesis provides an important basis for life on Earth because it drives the conversion of light energy into chemical energy that is used for building up complex organic material.

The first steps of plant photosynthesis mediate the photoinduced electron transfer from water to NADP+, catalyzed by Photosystems I and II (PSI and PSII) of the photosynthetic electron transfer chain. PSI and PSII are interconnected by the cytochrome *b_6_/f* complex, which transfers the electrons from PSII to PSI and translocates protons into the thylakoid lumen. This photosynthetic process, also termed “light reactions’, drives light-dependent water oxidation, NADP^+^ reduction, and ATP formation (Whatley et al. 1963). The formation of ATP is catalyzed by the ATP synthase driven by the proton-motive force generated by the light reactions (Mitchell 1961). ATP and NADPH are used by the light-independent “dark reactions’ of the Calvin–Benson–Bassham cycle (CBB) (Bassham et al. 1950) to assimilate CO_2_. During evolution, photosynthesis was optimized to increase photosynthetic performance while minimizing photo-oxidative stress and avoiding excess reactive oxygen species (ROS) formation. Several signaling mechanisms, including calcium (Ca^2+^) and redox signaling, balance the absorption of light energy with downstream energy-consuming pathways such as CO_2_ fixation and conversion (Foyer 2018; Hochmal et al. 2015). Once ROS are generated, several proteins relay this information to target proteins via redox signaling. At the same time, these proteins reduce and thereby detoxify ROS, thus commonly referred to as “ROS detoxifying” or “ROS processing enzymes” (Noctor et al. 2018). One important class of H_2_O_2_ interacting proteins within the chloroplast is peroxiredoxins (PRXs) (Dietz 2003; Poole et al. 2004; Dietz et al. 2006). These proteins harbor varying numbers of cysteines forming intramolecular disulfide bridges when oxidized by ROS. Interestingly, it has been recently discovered that PRXs, alongside their “antioxidative” action, can also act as sensor relays, transferring the oxidizing equivalents from H_2_O_2_ to their interacting partners and favoring the transmission of the oxidative stress response (Sobotta et al. 2015). Moreover, they are also involved in the oxidation of chloroplast enzymes in the dark (Ojeda et al. 2018). When oxidized, the regeneration of PRXs is accomplished by reduction via the thioredoxin (TRX) system (Pulido et al. 2010; Pascual et al. 2011) (Dietz 2011). TRXs use redox-active cysteines for target reduction while regaining their electrons from either reduced ferredoxin (Fd) via ferredoxin-TRX reductases or NADPH via NADPH-dependent TRX reductases. In the chloroplast of algae, plants, and some cyanobacteria, an atypical NTR with a TRX domain fused to its C-terminus can be found (Serrato et al. 2004). This enzyme, termed NTRC, functions as a homodimer (Pérez-Ruiz and Cejudo 2009) and is also able to directly reduce PRXs efficiently. Besides its antioxidant function (Perez-Ruiz et al. 2006), which has been shown to be of vital importance in Arabidopsis (*A. thaliana*), especially under low irradiance (Carrillo et al. 2016; Nikkanen et al. 2016), NTRC is involved in the redox regulation of a plethora of different metabolic pathways. These include the CBB cycle (Thormahlen et al. 2015), chlorophyll (Richter et al. 2013), and starch biosynthesis (Lepisto et al. 2013) and even fruit growth (Hou et al. 2019). The crystal structure of the *Chlamydomonas reinhardtii* (Chlamydomonas) NTR domain of NTRC was solved only recently (Marchetti et al. 2022).

Furthermore, Calredoxin (CRX), a TRX-like protein present in the green algae lineage and some Dinophyceae, was found to interact with the 2-cysteine peroxiredoxin PRX1 in *C. reinhardtii* (Hochmal et al. 2016; Charoenwattanasatien et al. 2020). CRX is a chloroplast-localized combination of a TRX with a Ca^2+^-binding domain, the latter controlling the redox activity of the TRX domain in vitro. It is involved in redox regulation and ROS scavenging after being induced by autotrophic and/or high light (HL) conditions (Hochmal et al. 2016). A decrease in CRX amounts in an insertional Chlamydomonas mutant (IM*_crx_*) led to increased lipid peroxidation and cyclic electron flow around PSI in vivo (Hochmal et al. 2016). As the redox potential of PRX1 is slightly more negative (−310 mV (Yoshida and Hisabori 2017)) than the redox potential of CRX (−290 mV (Hochmal et al. 2016)), reduction would be on the side of CRX, which by itself would drive partial oxidation of PRX1. However, electron transfer measured in vitro between CRX and PRX1 showed efficient reduction of PRX1, probably due to the negative potential of NADPH and the fast consumption via H_2_O_2_ reduction (Hochmal et al. 2016). Electron exchange between CRX and PRX1 in the one or other direction should therefore rely on the individual redox and Ca^2+^ status of the microenvironment. Interestingly, it was shown that in vitro electron transfer to PRX1 is similar for CRX and NTRC in the presence of Ca^2+^ (Marchetti et al. 2022). Notably, the NTRC midpoint redox potential was found at −275 mV (Yoshida and Hisabori 2016), which is slightly lower than that of CRX.

To gain further insights into CRX function, we performed in-depth quantitative proteomics analyses taking advantage of the *crx* insertional mutant (IM*_crx_*), two CRISPR-Cas9-generated knockout mutants (*crx* KOs, E1, and A5) and two complemented strains (IM-R and A5R). The data revealed a co-regulation of CRX and NTRC, underpinning the involvement of CRX in the chloroplast redox homeostasis of *Chlamydomonas*. The absolute quantitation of these two proteins and their use in in vitro redox assays interestingly highlighted an inhibitory role of CRX towards the general redox activity of NTRC and, in particular, towards its ability to reduce 2-Cys PRX1 in vivo. Taken together, our results highlight the possible role of CRX as a master regulator in the HL stress response in the chloroplast by regulating the expression and the activity of NTRC.

## Results

### Generation of *crx* KO mutants via CRISPR/Cas9 and phenotypic analyses

**Figure 1.**
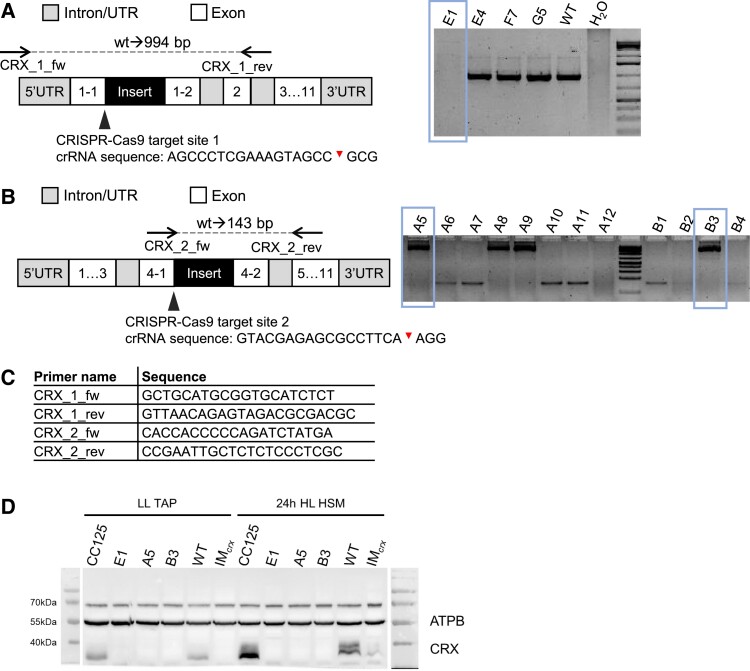
CRISPR-Cas9 mediated KO of CRX. Generation of CRX mutant affected in exon 1 **A)**. Only the clone, E1, showed a PCR product bigger in size than WT. A second set of mutants was created by targeting a sequence in exon 4 **B)**. Sequencing of PCR products deviating from WT product size showed that all respective mutants are isogenic, having part of the co-transformed paromomycin vector integrated after the predicted Cas9 cleavage site. Primer sequences used for mutant screening **C)**. For final proof of knockout, a chosen set of mutant and WT strains were subjected to a TAP/LL to HSM/HL (∼200 µEm^−2^s^−1^) shift. Cells were harvested, lysed and after separation by SDS-PAGE, proteins were transferred onto a nitrocellulose membrane and probed with antibodies against ATPB and CRX **D)**. As mutants A5 and B3 turned out to be isogenic while both are deficient in CRX, only mutant A5 was used for further experiments. Cas9 cleavage sites within the crRNA sequence are marked by red triangles in A and B.

To reveal insights into the function of CRX, two *crx* KO strains were generated from WT CC125 via CRISPR-Cas9 and were backcrossed three times with the parental strains CC124 (mt−) and CC125 (mt+) (Fig. 1). Importantly, these KO strains (E1 and A5) lacked residual CRX accumulation, unlike the previously described insertional CRX mutant IM_crx_, which showed about 10% to 20% CRX expression compared to the WT (Hochmal et al. 2016). The backcrossed A5 strain, as well as the IM_crx_, were complemented by transformation with a vector-harboring genomic DNA from 900 bp upstream until 300 bp downstream of the coding region in order to include regulatory motifs, such as the promotor region for *crx*. The complemented strains were selected for maximum accumulation of CRX by protein immunoblotting. For each *crx* mutant, one complemented strain with more than 50% CRX expression compared to WT was selected for further experimental analysis (A5-R and IM-R).

**Figure 2.**
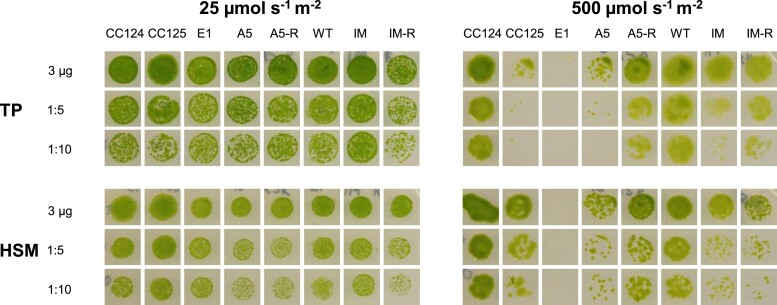
Lack of CRX leads to decreased growth in HL conditions. Growth performance of backcrossed CRISPR-Cas CRX mutants A5 and E1 and the insertional IM_crx_ was compared to the respective parental strains (CC124, CC125, and CC4375 (WT_crx_)) and rescues (A5-R and IM-R) by spotting 10 µl of culture at 3 µg/ml chlorophyll and the corresponding dilutions onto tris-phosphate (TP) or high-salt medium (HSM). Pictures were taken after 1 week of incubation at low (25 µmol m^2^ s^−1^) or high (500 µmol m^2^ s^−1^) light.

When examining the phenotypes, the first thing that became apparent was that wildtype, mutant, and complemented strains differed considerably in their ability to grow at HL intensities under photoautotrophic (tris-phosphate (TP) or high-salt medium (HSM)) growth conditions (Fig. 2). TP is more efficient in buffering pH changes than HSM and therefore has better control over the availability of nutrients (e.g. carbon), whereas HSM is considered to be the more “natural” growth medium (Glaesener et al. 2013). Different dilutions of the WT and backcrossed mutant and complemented strain cultures spotted onto agar plates showed comparable growth in low light (LL) but an impaired growth of the mutants compared to the respective wild types in HL. This effect is strongest for E1, followed by A5, whereas IM_crx_ is the least affected. The complemented strains showed an intermediate growth phenotype between respective *crx* mutants and WTs. The growth of WT CC125 was also affected in TP HL but not under HSM HL growth conditions.

### Quantitative proteomic analyses to identify proteins co-regulated with CRX

**Figure 3.**
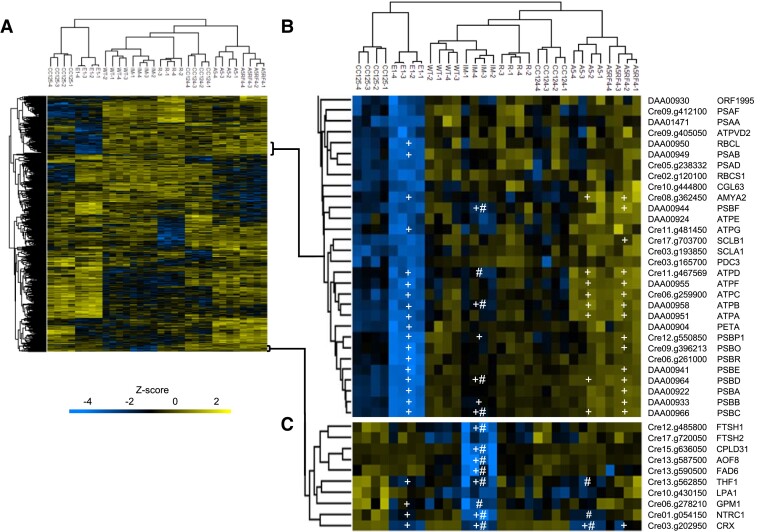
Lack of CRX induces decrease in NTRC accumulation. Hierarchical clustering of 881 proteins with significant differential abundance (*P* < 0.01, ANOVA followed by Tukey's post hoc test with FDR of 1%) after 24 h HL (500 μmol·m^−2^·s^−1^) **A)**. Downregulation of co-regulated proteins belonging to PSI, PSII, and ATPase in the E1 and CC125 strains **B)**. Proteins co-clustering with CRX **C)**. In the highlighted clusters, proteins with significantly different expression (*P* < 0.01) between either mutant/rescue vs corresponding wt or mutant vs rescue are indicated by + or #, respectively.

**Figure 4.**
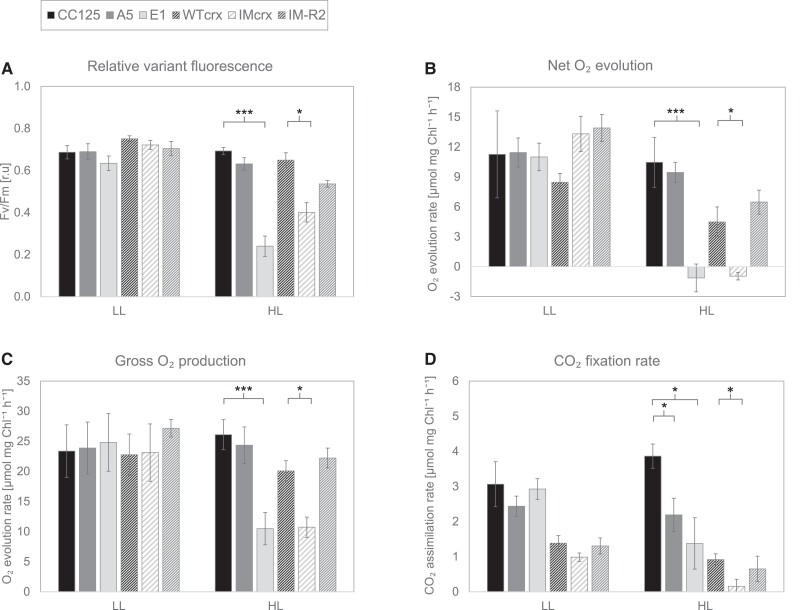
Decreased photosynthetic activity of *crx* mutants. CRISPR-Cas9 *crx* mutants A5 and E1, and the parental strain CC125 as well as WT_crx_, IM_crx_ and complemented strain IM-R2, were exposed to light following 12 h of low (30 µmol photons·m^−2^·s^−1^, LL) or high (500 µmol photons·m^−2^·s^−1^, HL) exposure. Quantum efficiency was determined by exposing the cells to a saturating pulse previous to illumination **A)**. Simultaneously, dissolved O_2_ and CO_2_ concentrations were evaluated by MIMS. O_2_ net evolution rate was determined by the increase of ^16^O_2_ concentration during light exposure **B)**. The gross O_2_ production rate was determined by subtracting the rate of ^18^O_2_ decrease in light (termed as “total uptake”) from the net O_2_ evolution **C)**. Gross CO_2_ fixation was determined by subtracting the rate of CO_2_ uptake in light from its increase under darkness **D)**. In all panels, columns represent averages of at least 5 biological replicates, with error bars indicating standard error. Statistical significance was determined using a Student's t-test, with asterisks indicating a *P*-value of * < 0.05 and *** < 0.001.

To unravel the underlying mechanisms of the CRX HL phenotype, the proteomes of the backcrossed CRX mutants, corresponding wild types and complemented strains were analyzed by mass spectrometry. For each strain, four independent biological replicates were measured. Synchronized cells were grown in TP medium in LL and then shifted to HL. Samples were taken at 0 (LL samples) and 24 h (HL samples) after the switch to HL. Whole protein was extracted from the samples, tryptically digested, and analyzed by HPLC-MS/MS. Out of 5,861 detected proteins, 881 were identified as differentially regulated in the different strains and submitted to hierarchical clustering (Fig. 3A). Among the clusters identified (Supplemental Table S1), those relevant to the phenotype are shown in detail in Fig. 3, B and C. Several proteins belonging to PSI and PSII, as well as ATPase, were found to be significantly downregulated in the strain E1 in comparison to the respective WT (CC125) (Fig. 3B), whereas these proteins were not affected or even upregulated in A5. Analyses of the growth phenotypes (Fig. 2) revealed that growth was strongly impaired in E1 and A5, but also of CC125 was more affected in HL TP, while in HSM, in contrast to E1, the growth of CC125 was not light sensitive. Interestingly, a significant downregulation of PSII and ATPase subunits is also observed in the IM_crx_ strain and rescued in the complemented IM-R strain (Fig. 3B). In line, PSII quantum efficiency and O_2_ evolution were severely affected in E1 and IM_crx_ but not A5 and IM-R (Fig. 4). Remarkably however, CO_2_ fixation in HL, as measured via membrane inlet mass spectrometry (MIMS), was affected not only in E1 but also in A5 and IM_crx_, pointing towards another phenotype, beside the growth defect, that is associated with the lack of CRX.

The other cluster of interest (Fig. 3C) contains all proteins co-regulated with CRX. c*rx* was significantly less expressed in all mutants, including IM_crx_, while the protein was significantly more abundant in the complemented strains, particularly in IM-R. The only partial re-establishment of *crx* expression in A5-R is in agreement with the spot test, where the growth performance of this strain was only partially restored. Interestingly, another protein involved in photosynthetic redox regulation belongs to this cluster, namely NTRC (Cre01.g054150). This protein has a pattern of expression highly similar to the one of CRX, being significantly less expressed in the *crx* mutants but re-established in the CRX-complemented strains. The other protein which showed a similar behavior is thylakoid formation 1 (THF1) (Cre13.g562850).

In conclusion, among the 881 differentially expressed proteins, given the distinct genetic backgrounds of E1, A5, and IM*_crx_*, only NTRC and THF1 expression patterns were found to correlate with CRX expression. This strongly suggests a functional link between these proteins and CRX. As NTRC is a hub of chloroplast redox regulation in vascular plants and a potential redox partner of CRX, we focused on the putative functional interconnection between these two proteins. The significant downregulation of NTRC in the *crx* mutants was also observed when quantitative proteomics data of all mutants were compared to all WTs via independent Volcano plot analyses (Supplemental Fig. S1A). It is also of note that the comparison of CC124 and CC125, as revealed by Volcano plot analyses, showed significant differences in protein expression, e.g. enzymes of acetate metabolism and a glutathione peroxidase were more abundant in CC124, while e.g. VIPP1, as well as ALD5, were more abundant in CC125 (Supplemental Fig. S1B).

### Redox activity measurements of electron transfer between CRX, NTRC, and PRX1

**Figure 5.**
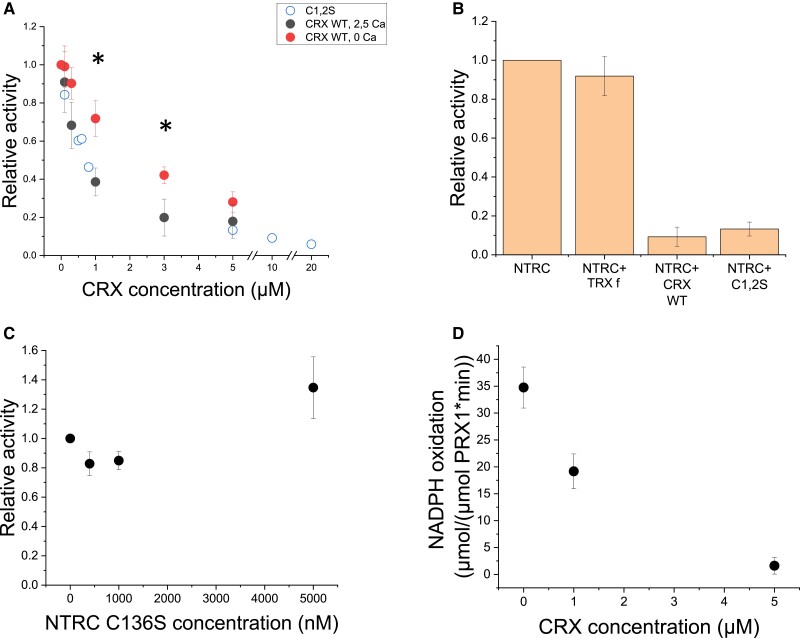
CRX specifically inhibits NTRC redox activity. 400 nM recombinant NTRC were incubated for 10 min at RT in presence of either 0 (black dots) or 2.5 µM (red and empty dots) free calcium, 200 µM NADPH, and different concentrations of either WT CRX (full dots) or its active site mutant (C1,2S, empty dots) **A)**. After 10 min 200 µM DTNB were added as substrate for reduction. The increase in absorption at 412 nm was recorded to calculate the redox activity (slope 0 to 80 s after addition of DTNB). Data were normalized on the highest activity measured for each protein purification. Error bars represent s.d. of three independent measurements. Stars indicate a significant difference by Student's t-test of *P* < 0.05. The same settings were used for incubation of 400 nM NTRC and 5 µM of either TRX f, CRX WT or its active site mutant (C1,2S) **B)**, and for incubation of 1 µM CRX, 200 nM TRXR and different concentrations of NTRC NTR domain active site mutant C136S **C)**. The reduction of 1 µM oxidized recombinant PRX1 was monitored by measuring NADPH oxidation at 340 nm in presence of 5 µM recombinant NTRC and different concentrations of CRX WT at a concentration of 2.5 µM free calcium in presence of 80 µM H_2_O_2_**D)**. In all panels, error bars represent standard deviation of three independent measurements.

As both proteins are known to function as oxidoreductases, electron transfer between the two proteins was analyzed in redox activity measurements (Fig. 5). Interestingly, the NTRC reduction capacity is negatively affected by CRX, as DTNB (Ellman's reagent) or PRX1 reduction is decreased with increasing concentrations of CRX (Fig. 5, A and D). This inhibition is independent of the CRX redox state, as also the redox-inactive CRX mutant C1,2S led to slower DTNB reduction. Furthermore, the lack of Ca^2+^ releases the inhibition partially (Fig. 5A), making Ca^2+^ an important compound for NTRC redox inhibition. The inhibition is specific to CRX, as shown by the fact that another redox-active protein, TRX f, had no negative impact on NTRC activity (Fig. 5B). On the contrary, the CRX reduction capacity was unaffected by the presence of NTRC-C136S, a mutated version with a redox-inactive NTR domain, but an active TRX domain, when electrons were donated by *E. coli* TRX reductase (Fig. 5C).

### Absolute quantitation of CRX and NTRC

**Figure 6.**
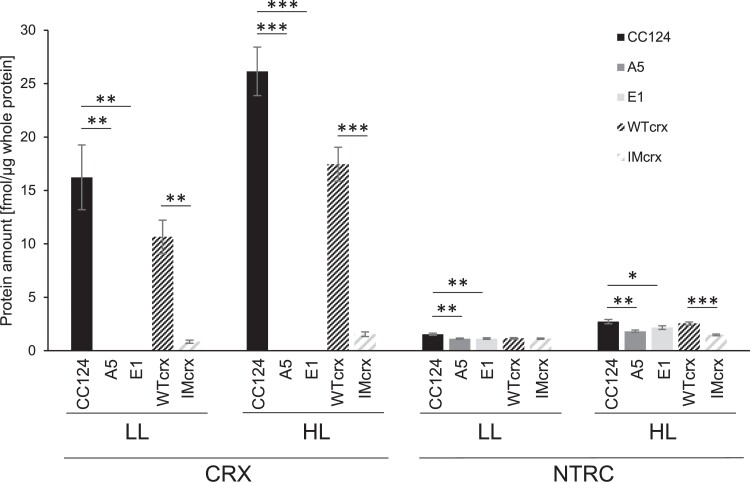
Determination of absolute CRX and NTRC amounts in the wild-type strains CC125 and wTcrx (CC4375) in LL (30 μmol·m^−2^·s^−1^) and HL (500 μmol·m^−2^·s^−1^). For absolute quantitation, recombinant ^15^N-labeled CRX and NTRC were spiked into ^14^N whole cell extracts grown in TP medium. Quantitation of intrinsic CRX and NTRC were based on known amounts of ^15^N-labeled CRX and NTRC. Bars indicate s.d. of 3 replicates with different amounts of ^15^N CRX/NTRC added. Stars indicates statistical significance by Student's t-test of * < 0.05, ** < 0.005, *** < 0.001.

To relate these functional properties of CRX and NTRC to a putative in vivo situation, absolute protein quantification was performed using protein samples obtained from algae cultivated in TP medium (Fig. 6). To do so, 50 μg of whole cell extract of TP-grown CRX WT or mutant cells were tryptically digested with 25, 125, or 500 fmol recombinant ^15^N-labeled CRX and NTRC. The different isotope masses allow us to distinguish between recombinant and endogenous CRX or NTRC and to relate the known ^15^N protein amounts to the unknown ^14^N protein amount from the cell lysate. As previously demonstrated by Hochmal et al. (2016), CRX expression was enhanced in the wild-type strains under high irradiance. Furthermore, no peptides corresponding to CRX were found in E1 and A5, confirming a successful mutation via CRISPR-Cas9 in these strains. In the WTs, CRX accumulation was much higher than NTRC, with an overall ratio of approximately 9 under both low and HL conditions. Furthermore, NTRC expression in the CRX mutants was significantly reduced in HL compared to the WTs, which is in line with results of the relative quantification (Fig. 3). Interestingly, this reduction was not present under low irradiance, where NTRC abundance was similar in all strains.

### Functional analyses of CRX and NTRC interactions

**Figure 7.**
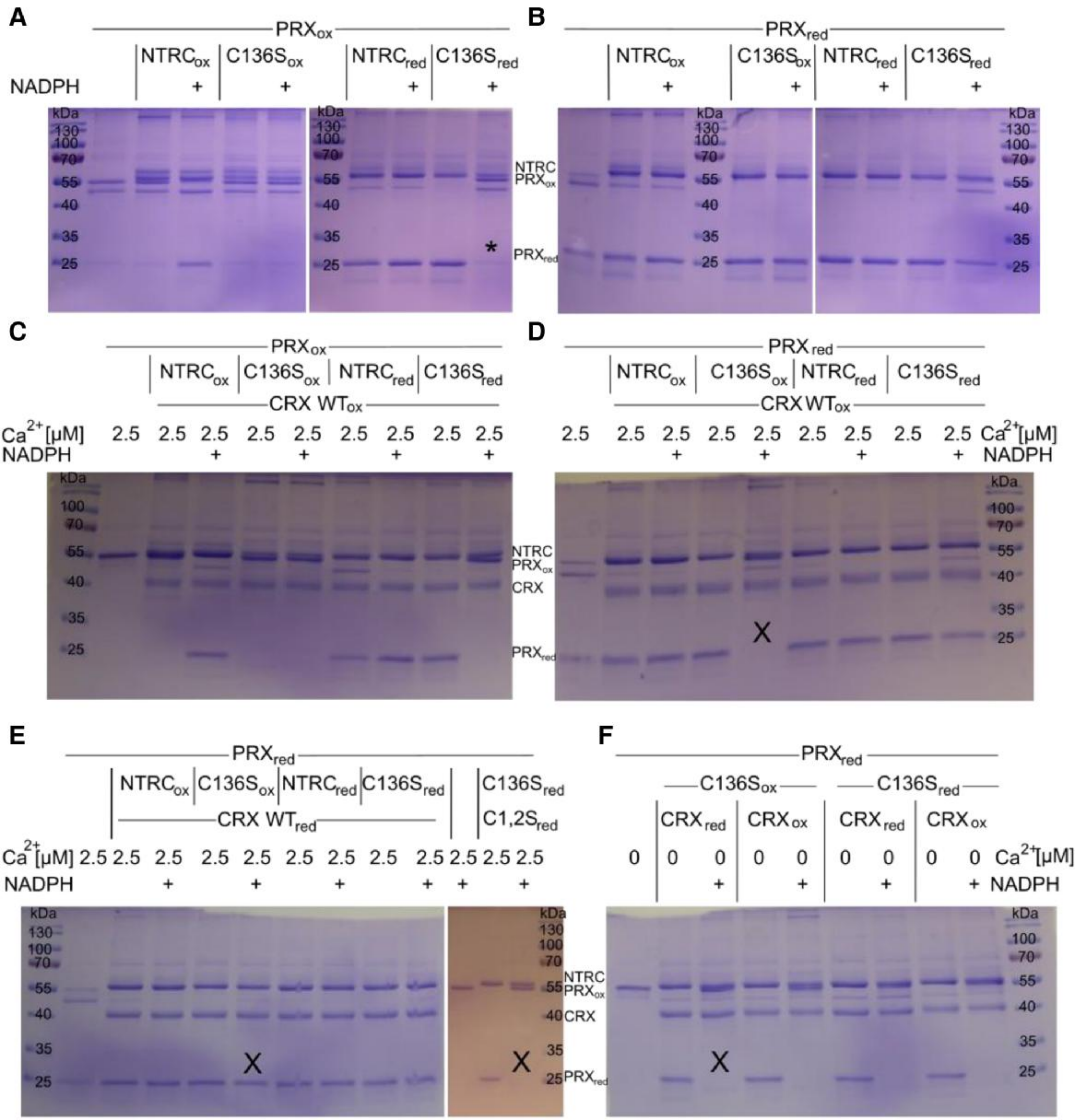
Oxidation of PRX1 is promoted via non-active CRX in the presence of NADPH. One microgram of purified recombinant PRX1 was reduced (oxidized) with 1 mM DTT (H_2_O_2_) for 1 h at room temperature (RT). After filtration over a G25 column, incubation with 1 µg of recombinant, equally reduced (oxidized) and filtered NTRC, NTRC-C136S, and/or CRX, CRX-C1 + 2S for 1 h at RT lead to redox exchanges between the different proteins. Presence or absence of Ca^2+^ and/or NADPH (100 µM) during this incubation is indicated. Before loading onto SDS-PAGE gels, addition of non-reducing loading dye and 20 min heating to 70°C ensured denaturation but allowed to distinguish between PRX1 dimers (PRX1_ox_) or monomers (PRX1_red_) after Coomassie staining. **A–F)** Results from two independent replicates. * and × denote important lanes referred to in detail in the text.

To analyze the NTRC-CRX interaction and its impact on the PRX1 redox state, non-reducing SDS-PAGE was performed after incubating the three proteins in different combinations and under varying conditions (Fig. 7). Because PRX1 is known to form dimers upon oxidation, the presence or absence of either monomer (∼25 kDa) or dimer (∼55 kDa) allows visualization of the PRX1 redox state. As long as CRX was absent, NTRC affected the PRX1 redox state as expected (Fig. 7, A and B): In experiments where reduced PRX1 was used as substrate, PRX1 stayed reduced, independent of the NTRC redox state and the presence of NADPH (Fig. 7B). When oxidized PRX1 was used as substrate, it remained oxidized as long as WT-NTRC and NADPH were added, which led to PRX1 reduction using electrons from NADPH (Fig. 7A). However, it could be reduced without NADPH when WT-NTRC or NTRC-C136S were already reduced before the experiment. Here, electrons from the reduced TRX domain of WT or NTRC-C136S could be directly transferred to PRX1 (Fig. 7A, lanes 8 to 10). In contrast, the NTRC-C136S mutant protein could not reduce PRX1 when NADPH was present (Fig. 7A, lane 11, marked with *), which led to the hypothesis that NADPH induces a conformational change making the TRX domain unavailable for PRX1 reduction in NTRC-C136S.

**Figure 8.**
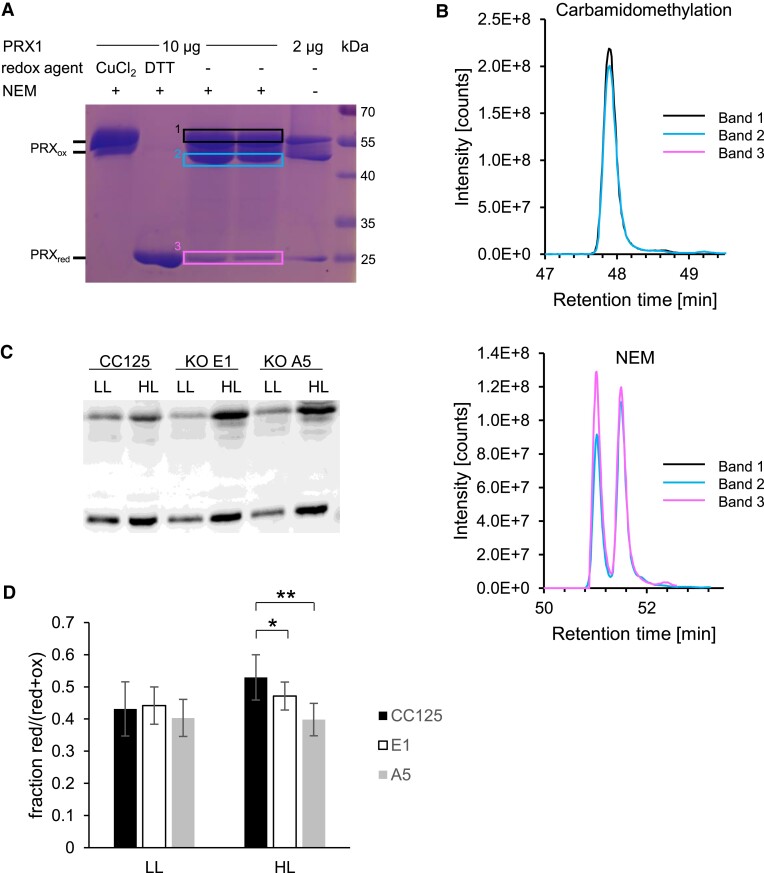
CRX is required to sustain reduction of PRX1 in vivo. Recombinant PRX1 was loaded onto a non-reducing SDS-PAGE after optional oxidation (50 µM CuCl_2_) or reduction (100 mM DTT) and NEM labeling **A)**. The two bands from dimerized PRX1 (bands 1 and 2) and the one representing monomeric PRX1 (band 3) were cut, reduced with DTT, carbamidomethylated and digested with trypsin. Peptides were analyzed by mass spectrometry as described in Materials and Methods. Panel **B)** shows precursor ion traces of the carbamidomethylated and NEM-labeled PRX1 peptide VLQAIQYVQSNPDEVCPAGWKPGDKTMKPDPK. The two peaks observed for the NEM-labeled peptide most likely represent two diastereomers, which were generated by introducing a new chiral center upon NEM labeling (Smyth et al. 1964; van den Hooven et al. 2001; Esteban-Fernández et al. 2011). *In vivo* PRX1 redox state in CRX WT and mutant strains before (LL) and after (HL) a 24 h shift to 500 µEm^−2^s^−1^**C)**. Samples were harvested in 10% (w/v) TCA to block the thiol redox state and subsequent NEM labeling avoided reoxidation during sample handling. Samples were run on a non-reducing SDS-PAGE and blotted onto a nitrocellulose membrane. PRX1 was detected by a polyclonal antibody raised against the recombinant PRX1. The fraction of reduced PRX1 (intensity reduced band(s) divided by the sum of reduced and oxidized bands within one sample as measured by ImageJ) was calculated and plotted in **D)**. Error bars represent standard deviation of 6 independent experiments. Stars indicate significant differences by Student's t-test with a *P*-value of * < 0,05 and ** < 0,005.

When oxidized CRX was added, the same results were obtained (Fig. 7, C and D), except for the NTRC-C136S mutant, in the presence of NADPH and previously reduced PRX1 (Fig. 7D, lane 5, marked with X). Under these conditions, PRX1 appeared to be oxidized when oxidized CRX is present, suggesting that in the presence of NADPH, either CRX binding makes the oxidized TRX domain of NTRC-C136S available for PRX1 oxidation or that the oxidized CRX itself oxidizes PRX1. However, when reduced CRX was added, this oxidation was probably overcome by re-reduction via CRX (Fig. 7E, lane 6, X), as previously reported (Hochmal et al. 2016). As CRX is known to be active only in the presence of Ca^2+^ (Hochmal et al. 2016), the addition of EGTA abolished this re-reduction, and PRX1 became oxidized even in the presence of reduced CRX (Fig. 7F, lane 3, X). Also, the use of reduced CRX double cysteine mutant (C1 + 2S) did not lead to PRX1 re-reduction (Fig. 7E, lane 13, X), underpinning the importance of redox-active CRX for reduction of PRX1. In conclusion, the simultaneous presence of inactive CRX (oxidized, mutated active Cys, or absence of Ca^2+^) and NADPH impacted PRX1/NTRC in a way that PRX1 did not stay reduced. Importantly, the lack of Ca^2+^ in the presence of NADPH always led to PRX1 oxidation, independent of the redox state of NTRC-C136S or CRX (Fig. 7F). As the NTRC-C136S mutant cannot reduce its own TRX domain, electron replenishment from NADPH is disabled. Therefore, oxidation of PRX1 can be observed instead of being overruled by re-reduction via WT-NTRC. As PRX1 is the main CRX/NTRC target, non-reducing gels after NEM labeling were run and analyzed for the fraction of reduced PRX1 in vivo (Fig. 8). Indeed, we observed a significant disability to keep PRX1 in a reduced state in the *crx* KO mutants A5 and E1 in comparison to WT.

**Figure 9.**
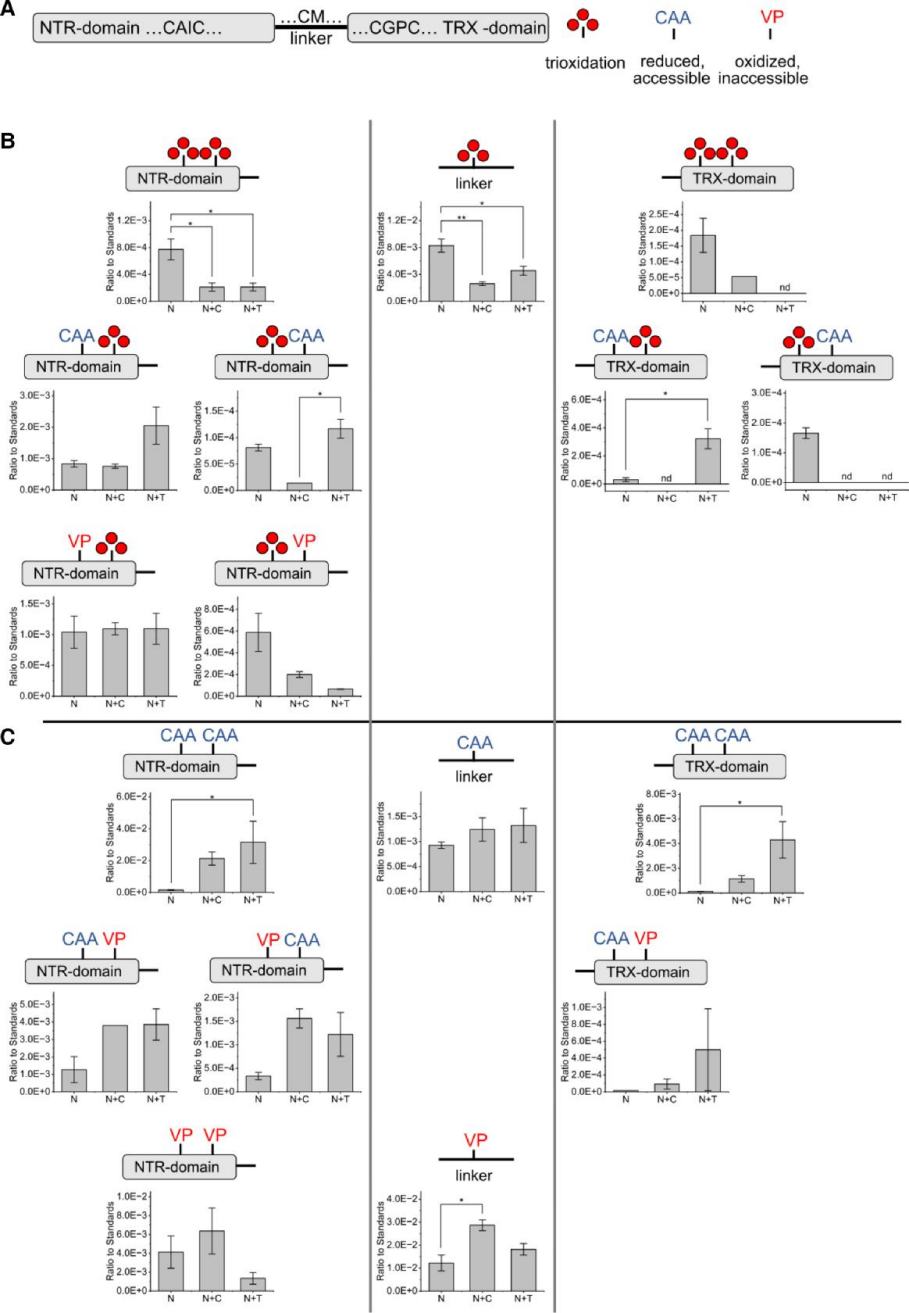
Redox modifications of NTRC cysteines upon incubation with or without CRX or TRX f. NTRC (N) was incubated alone or with 10 times more CRX (N + C) or TRX f (N + T) in Ca^2+^-free MOPS/KCl buffer containing NADPH. Reduced and accessible cysteines were labeled with chloroacetic acid (CAA). After removal of residual CAA and subsequent TCEP reduction, 2-vinylpyridine (VP) marked previously oxidized or inaccessible cysteines. After tryptic digestion, modifications of NTRC cysteine peptides (GISACAICDGASPLFK, NTR domain; QAITAAGSGCMAALSAER, linker; LICVLYTSPTCGPCR, TRX-domain) were quantified by parallel reaction monitoring. Scheme of possible NTRC cysteine modifications **A)**. Peak areas of peptides with at least one trioxidated cysteine **B)**. Peak areas of remaining cysteine peptides including CAA and VP modifications **C)**. In all panels, each column represents the average of three replicates with error bars displaying the standard error of the mean. For normalization between replicates, peak areas are shown as ratios to the summed peak areas of two NTRC peptides lacking cysteines (ANLKPVVFEGFR, LVAGQVELDEAGYVK). Statistical significance was determined by one-way ANOVA followed by Tukey's honest significance test. Asterisks indicate *P*-values of * < 0.05, ** < 0.01 and *** < 0.001. nd = not detected.

In order to further elucidate the mechanism of NTRC inhibition by CRX, we employed redox proteomics to investigate the redox state of NTRC cysteines in the presence or absence of CRX or TRX f (Fig. 9). We observed that the NTRC cysteines were quickly inactivated via trioxidation when NTRC was incubated with NADPH in the absence of CRX or TRX f. Addition of CRX (and partially also TRX f) prevented this trioxidation as seen by lower peak areas of the respective trioxidated peptides (Fig. 9B). During the experiment, the remaining free thiols were labeled with chloroacetic acid (CAA), representing the reduced cysteines. Because labeling was done under native conditions, carbamidomethylation also indicates accessibility of the cysteines by the alkylating agent. After subsequent reduction and denaturation, vinylpyridine (VP) labeling marked the previously oxidized or inaccessible cysteines. Due to the strong trioxidation in the samples containing only NTRC, CAA-, and VP-labeled peptides were less present as compared to the samples containing also CRX or TRX f (Fig. 9C). When CRX and NTRC were incubated together, NTRC cysteines were found rather oxidized (VP), whereas incubation with TRX f led to significantly more reduced (CAA) NTRC cysteines (Fig. 9C). Generally, the peptides from the NTR-, linker-, or TRX-domain behaved similarly with regard to oxidation status, albeit especially for the samples containing CRX; not many peptides of the TRX domain could be quantified.

## Discussion

To shed further light on CRX function, we used two CRX CRISPR/Cas9 knockout mutants and an insertional mutant, as well as CRX-complemented strains. Analysis of these strains via (relative) quantitative proteomics pointed to a functional link between CRX and NTRC (Figs. 3 to 9). This functional link was further demonstrated by combined enzyme and non-reducing gel assays. Moreover, the data revealed a severe HL growth phenotype in CRX-depleted strains (Figs. 1 and 2), as well an impact on CO_2_ fixation under HL conditions (Fig. 4).

### Quantitative proteomics reveals co-regulation of CRX and NTRC

Using mass spectrometry and subsequent hierarchical clustering of protein accumulation profiles, CRX was found to be co-regulated with NTRC and THF1 (Fig. 3C). THF1 is known to be involved in stabilization and assembly of the FTSH subunits in Arabidopsis (Zhang et al. 2009) and Synechocystis (Bec Kova et al. 2017), which are essential for the D1 repair cycle and therefore HL acclimation. In our data, FTSH1 and 2 also cluster together with CRX, albeit FTSH1 is significantly downregulated only in IM_crx_ and not in the knockout mutants (Fig. 3C). Concerning FTSH2, a similar trend as for CRX is evident. In the independently analyzed volcano plots, FTSH1 could be shown to be significantly less abundant in all *crx* mutants than all WTs (Supplemental Fig. S1A). The strongly impaired ability of CRX mutants to grow in HL conditions, therefore, may also be partly due to a defect in photosystem II repair, at least in IM_crx_ and E1, similar to the THF1 phenotype in Arabidopsis (Sakamoto et al. 2002). Accordingly, PSII proteins were significantly lower abundant in IM_crx_ and E1 than in the respective WTs. However, the A5 mutant did not show a decrease in PSII as compared to CC124 (Fig. 3B), thus indicating that the PSII phenotype does rather depend on genetic background differences than solely on CRX depletion. Also, other proteins such as RuBisCO and chloroplast ATP synthase (Fig. 3, Supplemental Table S1) were decreased in IM_crx_ and E1 but not in A5, again pointing to differences that manifested in the absence of CRX, as these phenotypes were recovered in the complemented strains, but varied due to the genetic background of these strains. These differences in genetic compositions are consistent with the extensive natural variations found in *Chlamydomonas* (Flowers et al. 2015). Differences in protein expression were also revealed between CC124 and CC125 (Supplemental Fig. S1B). In this light, CRX is a capacitor that buffers genetic variations, contributing to phenotypic robustness as described for HSP90 (Queitsch et al. 2002).

### CRX inhibits electron transfer between NTRC and PRX1

As NTRC is also a thioredoxin-like protein and, as such, harbors two pairs of redox-active cysteines, we assessed electron transfer between NTRC and CRX, as well as PRX1 (Fig. 5). Our data revealed that NTRC and CRX did not efficiently exchange electrons. Instead, the presence of CRX inhibited electron transfer from NTRC to PRX1. The degree of inhibition was positively correlated with the amount of CRX added. Considering the amounts of NTRC and CRX as determined by absolute protein quantification in whole cells (Fig. 6), NTRC should be mostly inhibited by CRX in HL conditions, assuming the two proteins are in close vicinity to each other inside the chloroplast. The NTRC inhibition by CRX was independent of the CRX redox-active site and, therefore, cannot be attributed to a direct interaction between the redox-active cysteines of NTRC and CRX. Yet, the presence of Ca^2+^ ions had a significant impact on the inhibitory action of CRX towards NTRC (Fig. 5). Ca^2+^ is known to activate the CRX redox activity through a conformational change stretching from the Ca^2+^-binding domain to the redox domain of CRX (Charoenwattanasatien et al. 2020). According to the data presented here, this conformational change could, in fact, not only lead to CRX activation but also allow reversible binding of CRX to NTRC (or PRX1) at a site so far unknown to regulate the NTRC redox activity towards PRX1. A change in Ca^2+^ concentration within the chloroplast stroma would therefore impact the redox signaling network not only at the level of CRX but also indirectly via CRX at the signaling hub NTRC.

Taking advantage of the C136S mutant of NTRC harboring an inactive NTR domain (Marchetti et al. 2022) allowed us to further dissect the NTRC-CRX-PRX1 interaction (Fig. 7), as NADPH-dependent re-reduction of the still active NTRC-TRX domain is disabled in this mutant. We observed a reduction of oxidized PRX1 by reduced NTRC and in the presence of NADPH as expected, except for the sample containing NADPH and reduced NTRC-C136S. Although reduced NTRC-C136S alone was able to reduce oxidized PRX1, the additional presence of NADPH inhibited this PRX1 reduction (Fig. 7A). As suggested by Marchetti et al., the binding of reduced NADPH could lead to a conformational change within the NTRC protein to adopt a “closed conformation” and protect its reducing power from the oxidizing environment. As NADPH cannot be oxidized in NTRC-C136S, this “closed conformation” might be stabilized so that electron transfer between the TRX domain of NTRC-C136S and PRX1 is not possible when NADPH is added. Additionally, it should be noted that NADPH leads to dimerization, and thereby activation, of NTRC by breaking up protein aggregates formed under oxidizing conditions (Pérez-Ruiz and Cejudo 2009).

Once PRX1 was reduced, NTRC and NTRC-C136S were both able to stabilize it and inhibit PRX1 dimerization upon the addition of NADPH (Fig. 7B). In the presence of redox-inactive CRX, however, PRX1 dimerization was favored in the presence of NADPH (Fig. 7, D to F). Upon addition of reduced CRX in the presence of Ca^2+^, PRX1 remained reduced, as reported by Hochmal et al. (2016). The finding that NTRC-C136S cannot stabilize reduced PRX1 in the presence of NADPH and CRX (Fig. 7, D to F) underpins a direct protein–protein interaction either between NTRC-C136S and CRX, which induces a conformational change that does not further allow to stabilize reduced PRX1, or between CRX and PRX1, which prevents NTRC from acting on PRX1.

Our redox proteomic data propose a direct interaction between NTRC and CRX, as the addition of CRX diminishes the trioxidation of NTRC cysteines (Fig. 9). Because differences in VP and CAA labeling between samples containing CRX or TRX f were also observed, the impact of CRX on NTRC seems to be unique to this protein. As TRX f led to more CAA labeling in relation to the control NTRC than CRX did (Fig. 9C), the NTRC NTR and TRX domain cysteines seem to be more reduced in the presence of TRX f or are more readily accessible for labeling in the native conformation than when CRX was present. This difference is in line with the observation that the addition of CRX but not TRX f impacted the enzymatic activity of NTRC (Fig. 5B). Moreover, the diminishment of NTRC peptides harboring CAA-labeled cysteines in the presence of CRX would indicate that CRX binding to NTRC impacts the reduction of the NTRC active cysteines either directly or indirectly via making them accessible for redox modification.

**Figure 10.**
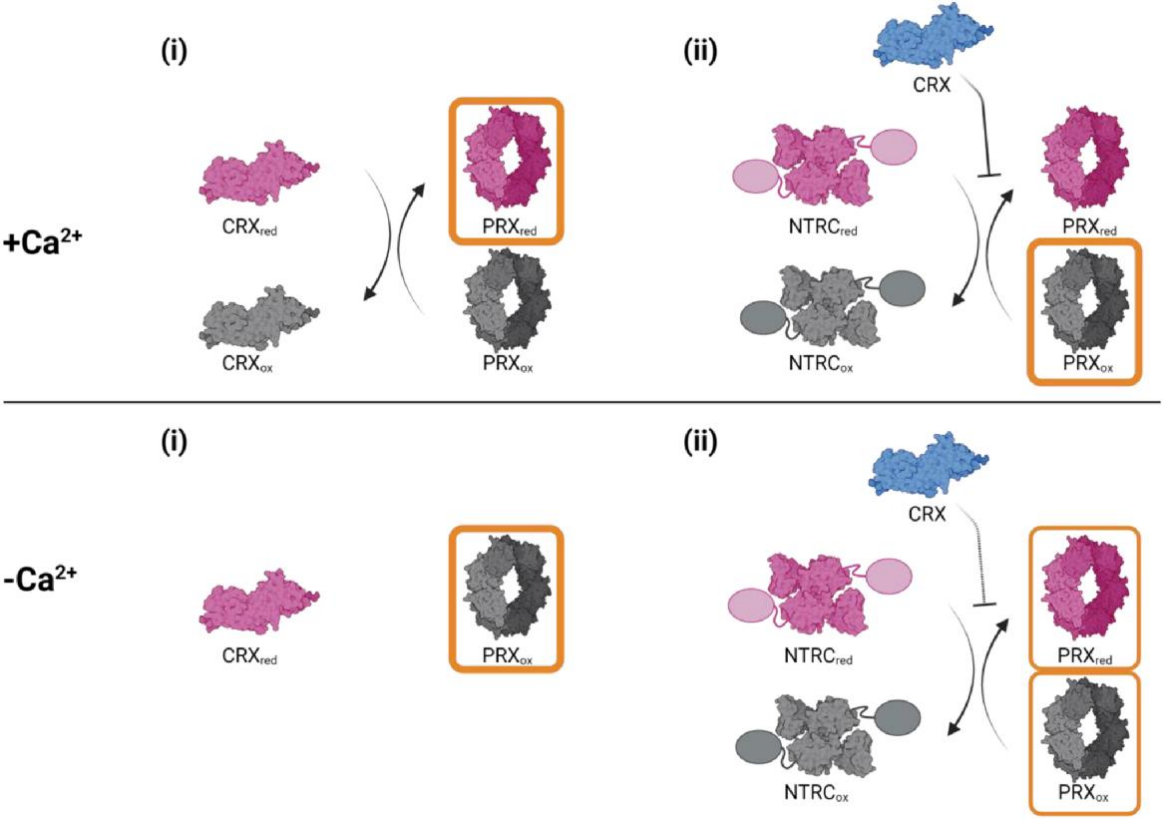
Model for CRX, PRX1, and NTRC interaction in the presence of NADPH. In the presence of Ca^2+^, CRX can reduce PRX1 (i) and also inhibits NTRC reduction of PRX1 (ii). In the absence of Ca^2+^, CRX cannot reduce PRX1 and NTRC inhibition is weaker. The resulting redox state of PRX1 is marked in orange. Figure created with BioRender.com.

These results would imply a dual role of CRX for PRX1 reduction inside the chloroplast, illustrated schematically in Fig. 10: In the presence of Ca^2+^, it might (i) directly reduce oxidized PRX1, thereby getting oxidized itself and (ii) inhibit NTRC-driven PRX1 reduction in the presence of NADPH by direct protein–protein interaction (Fig. 7, D to F). The CRX-dependent inhibition of PRX1 reduction by NTRC was also valid at low Ca^2+^ (Fig. 7F), albeit at a lower intensity (Fig. 6). Accordingly, inactive CRX would lead to a more oxidized pool of PRX1s, as it cannot reduce PRX1 itself, plus it prevents reduction by NTRC in the presence of NADPH.

The function of CRX would be particularly important under conditions where cellular Ca^2+^ spikes in response to various biotic and abiotic stresses are evoked (see Kudla et al. (2018) and Pirayesh et al. (2021) for recent reviews on Ca^2+^ signaling in plants) because the physiological Ca^2+^ concentration in the stroma, which is at about 100 to 150 nM Ca^2+^ (Johnson et al. 1995), is lower than the Ca^2+^ concentration needed for half-maximal CRX electron transfer rate (about 280 nM free Ca^2+^) (Hochmal et al. 2016). In the presence of elevated stromal Ca^2+^ concentrations, CRX could be reduced via an NADPH-driven NTR-like reductase, allowing CRX to drive PRX1 reduction (Hochmal et al. 2016). Evidence for CRX-driven PRX1 reduction is attained by the fact that in HL, PRX1 levels are significantly more oxidized in the CRX KO mutants than in the WT (Fig. 8).

However, keeping in mind that the redox potentials of CRX (−280 mV) and PRX1 (−310 mV) are similar, CRX reduction via PRX1 should also be possible. The oxidation of PRX1 via CRX in the presence of Ca^2+^ (Fig. 7D) could be explained accordingly, also because NTRC-C136S stabilized reduced PRX1 in the absence of CRX (Fig. 7B), although its NTR domain is not redox active anymore. As mentioned before, the overall CRX reduction level would also critically depend on the stromal Ca^2+^ concentration.

Under conditions where PRX1 gets quickly oxidized (e.g. stresses like HL, heat, and ROS burst), the reduced CRX pool would drive rapid re-reduction of PRX1. In this scenario, CRX could be regarded as a Ca^2+^- regulated redox buffer, important for PRX1 redox status control. At the same time, oxidized CRX could also drive PRX1 oxidation and impact PRX1 reduction via NTRC in the presence of NADPH shortly after PRX1 has accepted electrons from CRX and the Ca^2+^ spike is over. This scenario, in which CRX is a main player, would therefore be important to return to a steady-state redox equilibrium after transient stress signaling. In addition, under constant high stromal Ca^2+^, when CBB cycle activity is slowed down, as in the transition from light to dark (Hochmal et al. 2015), CRX-mediated inhibition of NTRC would further diminish CBB activity because it would render the redox state of key CBB enzymes to be more oxidized.

Binding of CRX to NTRC or NTRC-C136S in the presence of NADPH (Fig. 7, D to F) induced a conformational change that impacts the reduction and redox state of PRX1. Notably, the catalytically active form of NTRC is the dimer, whose formation is induced by NADPH (Pérez-Ruiz and Cejudo 2009). In this way, the function of the NADPH-NTRC complex appears to be under the strict control of CRX. The involvement of NADPH in the PRX1 redox tuning via CRX and NTRC allows interconnecting of the redox state of the three proteins with active photosynthetic electron transport and metabolism, thereby ensuring the economical use of NADPH for PRX1 reduction. In this light, we propose that the Ca^2+^-dependent redox equilibrium of CRX and PRX1 also depends on the availability of NADPH, where CRX additionally suppresses the reduction of PRX via NTRC.

This situation appears to be different from *Arabidopsis thaliana*, where CRX is absent, and NTRC is the most potent electron donor to reduce the oxidized disulfide form of PRX1 (Pulido et al. 2010). In Chlamydomonas, CRX not only controls electron transfer from NTRC towards PRX1 but can also reduce PRX1 efficiently and is about 10-fold more abundant than NTRC. Thus, we suggest for Chlamydomonas that in the presence of NADPH, CRX is the main electron donor for PRX1 reduction under HL, where CRX is also induced in expression. In darkness or LL, NTRC would be fully functional.

In plants, the absence of NTRC causes growth phenotypes under various abiotic stresses (Serrato et al. 2004; Perez-Ruiz et al. 2006; Chae et al. 2013), particularly under fluctuating light HL regimes (Naranjo et al. 2016; Thormahlen et al. 2017; Guinea Diaz et al. 2020). It was also shown that decreased levels of chloroplast 2-Cys PRX suppress the phenotype of the *A. thaliana ntrc* KO, indicating that NTRC is involved in the redox balance of chloroplast 2-Cys PRX and optimal photosynthetic performance (Pérez-Ruiz et al. 2017). In Chlamydomonas, in the absence of CRX, we also observed a more oxidated state of PRX1 (Fig. 8), thus suggesting that CRX, in conjunction with NTRC, is likely involved in redox balance of chloroplast PRX1 and the optimal function of the photosynthetic apparatus. This is exemplified in a strong impact on CO_2_ fixation, which is manifested in IM_crx_ as well as in the two *crx* KO strains (Fig. 4) and the strong HL phenotype observed here (Fig. 2). Thus, these data indicate that CRX is involved in chloroplast redox modulation of TRX targets, besides its redox regulation of PRX1. If, however, this impact is exerted mainly directly via CRX redox activation/deactivation or indirectly via control of NTRC redox activity is difficult to distinguish. Extensive comparative analysis of *ntrc* single mutants could help to solve this question. The observation, that CRX impacts chloroplast CO_2_ fixation under HL indicates the unique importance of CRX for the robustness of the system. In line, neither NTRC accumulation nor the growth of CRX mutants is affected under low irradiances (Figs. 2 and 6). Despite the different protein profiles of the CRISPR/Cas9 and insertional mutant, the strong HL growth phenotype is particularly obvious in the CRISPR/Cas9 mutants and rescued in the A5 complemented strain.

Thus, in conclusion, the KO of CRX is responsible for the HL and CO_2_ fixation phenotypes, possibly linked to elevated ROS formation as described (Hochmal et al. 2016) but not to the PSII decrease in the *crx* KO E1. The impact of the genetic WT background, as already discussed, is striking and indicates that, in general, observed phenotypes have to be taken with caution, especially when regulatory proteins are knocked-out but also when gene rescue approaches are undertaken. For example, in the IM_crx,_ PSBD and PSBC are significantly diminished in HL, yet, rescued in IM_crx_R2. However, PSBD and PSBC are not impacted in expression in the KO A5 under HL (Supplemental Table S1).

In summary, our investigations revealed a so far unknown novel role of CRX in Chlamydomonas redox homeostasis, as it impacts NTRC function towards PRX1, adding another layer of complexity to the chloroplast redox and signaling network. This CRX-dependent chloroplast redox regulation is essential for efficient photosynthesis in *Chlamydomonas*.

## Materials and methods

### Strains and culture conditions

The IM_crx_ with residual CRX accumulation was generated previously (Hochmal et al. 2016) from Chlamydomonas (*C. reinhardtii*) strain CC-4375 (designated as WT_crx_ throughout this article). The mutants E1 and A5, affected in exon 1 and exon 4, respectively, were generated via the CRISPR-Cas9 method from the wild-type strain CC125 (mt+) as described before (Greiner et al. 2017; Kelterborn et al. 2022), to obtain complete *crx* knock-outs (KOs). Primers used for mutant screening are listed in Fig. 1C. These strains were then crossed three times with the CC124 (mt−). Progenies without CRX accumulation, as evidenced by protein immunoblotting, were crossed back into CC125 (mt+), checked again and crossed a third time into CC124 to generate mutants with a clear genetic background. Expression of *crx* was partially restored in the strains IM-R, IM-R2, and A5-R. This was done by introduction of the endogenous *crx* gene (harboring only the first intron), including additionally 900 bp upstream (putative promotor region) and 300 bp downstream (possible additional region important for expression regulation) of the gene to IM_crx_ and A5, respectively. The construct was delivered together with a zeocine resistance cassette to screen for successfully transformed mutants that incorporated the gene into their genome. Induction of expression and expression level was checked by protein immunoblotting, and mutants with the highest expression level were selected for further analysis.

Strains were grown at 25°C and 120 rpm photo-heterotrophically in tris-acetate-phosphate (TAP) medium or in tris-phosphate (TP) medium to induce photoautotrophic growth. Light intensities were low (25 to 40 μEm^2^s^−1^, LL) or high (200 to 500 μEm^2^s^−1^), as indicated.

### Growth test

Cells were grown under LL in TP medium for three days until a chlorophyll concentration of 37g/ml was reached. Ten microliters of these cultures were spotted onto two TP or HSM plates each, together with the same volume of culture diluted as indicated. One plate was placed in LL, while the other was incubated under HL (500 µE m^−2^ s^−1^).

### Sample preparation for whole proteome analysis

Cells were grown at 40 µmol photons·m^−2^·s^−1^ (16 h light/8 h dark) for 3 days in TP and were then adjusted to 4 μg/ml chlorophyll in fresh TP after approximately 4 h in the light cycle. Immediately afterwards, LL samples were harvested, and the remaining culture was shifted to HL (500 μEm^−2^s^−1^) for 24 h. Depending on the culture concentration, 10- to 20-ml samples were taken at 0 or 24 h HL by centrifuging at 2500*×g* for 5 min at room temperature. Pellets were frozen in liquid nitrogen and stored at −80°C until use. Thawed pellets were resuspended in a 1:5 pellet:lysis buffer ratio (150–300 μl, 100 mM Tris–HCl, pH 8, 2% (w/v) SDS, 10 mM NaF, 10 mM sodium pyrophosphate, 10 mM β-glycerophosphate, 1 mM Na-orthovanadate, 1 mM PMSF, 1 mM benzamidine), heated to 65°C for 20 min while shaking at 1000 rpm, sonicated for 5 min in a sonication bath and centrifuged at 14,000*×g* for 10 min. The protein concentration of the supernatant was determined by bicinchoninic acid assay (Pierce BCA Protein Assay Kit, Thermo Fisher Scientific). One hundred micrograms of protein were loaded into Filter-Aided Sample Preparation (FASP) filters (Amicon Ultra 30 K, 0.5 ml, Merck) and tryptically digested overnight following the FASP protocol as described in Wśsniewski et al. (2009) with the following modifications: dithiothreitol (DTT) and iodoacetamide were replaced by tris(2-carboxyethyl)phosphine) (TCEP) and chloroacetamide (CAA), respectively, allowing reduction and alkylation of cysteines in a single step.

Prior to MS analysis, aliquots corresponding to 5 μg of digested peptides were desalted on C18 membranes as described (Rappsilber et al. 2003), dried by vacuum centrifugation and stored at −80°C until further use. Prior to LC-MS/MS analysis, samples were reconstituted in 5 µl of 0.05% (v/v) trifluoroacetic acid (TFA)/4% (v/v) acetonitrile (ACN).

### LC-MS/MS analysis of whole proteome

Sample analysis was carried out on an LC-MS system consisting of an Ultimate 3000 nanoLC (Thermo Fisher Scientific) coupled via a Nanospray Flex ion source (Thermo Fisher Scientific) to a Q Exactive Plus mass spectrometer (Thermo Fisher Scientific).

Approximately 1 µg of peptides were loaded on a trap column (C18 PepMap 100, 300 µM × 5 mm, 5 µm particle size, 100 Å pore size; Thermo Fisher Scientific) using loading buffer (0.05% (v/v) trifluoroacetic acid (TFA)/4% (v/v) ACN in MS-grade water) for 3 min at a flow rate of 10 µl/min. Peptides were separated on a C18 column (PepMap 100, 75 µm × 50 cm, 2 µm particle size, 100 Å pore size; Thermo Fisher Scientific) at a flow rate of 250 nl/min. The eluents were 0.1% (v/v) formic acid (FA) in MS-grade water (A) and 0.1% (v/v) FA/80% (v/v) ACN in MS-grade water (B). The following gradient was applied: 5% to 24% B over 120 min, 24% to 36% B over 40 min, 36% to 99% B over 10 min, 99% B for 20 min. MS data were collected by data-dependent acquisition (DDA), dynamically choosing the 12 most abundant ions from the precursor scans (scan range *m/z* 350 to 1,400, resolution 70,000, AGC target value 3e6, maximum injection time 50 ms) for fragmentation (MS/MS) by higher-energy C-trap dissociation (27% normalized collision energy, isolation window 1.5 *m/z*, resolution 17,500). AGC target value for MS/MS was 5e4 at 80 ms maximum injection time and an intensity threshold of 6.9e3. Dynamic exclusion was enabled with an exclusion duration of 60 s. Singly charged ions, ions with charge state 5 and above, as well as ions with unassigned charge states, were excluded from fragmentation. Internal lock mass calibration was enabled on *m/z* 445.120025.MS raw files were converted to mzML format employing ThermoRawFileParser (version 1.3.3, (Hulstaert et al. 2020). Data were analyzed with MSFragger 3.3 (Kong et al. 2017), Philosopher 4.0 (da Veiga Leprevost et al. 2020), and IonQuant 1.7.5 (Yu et al. 2020), all implemented in Fragpipe (version 16.0). The Fragpipe profile “LFQ-MBR” was used with default settings for protein identification and label-free quantification. Spectra were searched against a *C. reinhardtii* protein sequence database consisting of nuclear-encoded (www.phytozome-next.jgi.doe.gov, assembly version 5.0, annotation version 5.6), chloroplast-encoded (NCBI BK000554.2), and mitochondria-encoded (NCBI NC_001638.1) proteins. Common contaminants (cRAP, www.thegpm.org/crap/) were also included. Carbamidomethylation was set as a fixed modification. Oxidation of methionine and acetylation of protein N-termini were considered variable modifications.

LFQ data (combined_protein.tsv) were loaded into Perseus 1.6.15.0 (Tyanova and Cox 2018) for processing and statistical analysis. After log2-transformation, proteins with less than three valid intensity values in at least one strain were filtered out. Then, intensity data from strains with identical genetic backgrounds were grouped, and proteins not meeting a threshold of 75% of valid values in at least one group were discarded. CRX was exempted from filtering. Remaining missing values were imputed from a normal distribution (width: 0.3, down shift: 2.5). Subsequently, multiple sample testing (ANOVA) was performed with a permutation-controlled false discovery rate (FDR) of 0.01, followed by Tukey's honestly significant difference test (FDR 0.05). After z-scoring of protein intensities, hierarchical clustering (average linkage, Euclidean distance, 30 clusters) of significantly differentially expressed proteins was carried out. Volcano plots were generated using LFQ-Analyst (Shah et al. 2020), which uses the Limma (Ritchie et al. 2015) package for differential expression analysis. Imputation of missing data was omitted in LFQ-Analyst since this step was already performed in Perseus. FDR correction was carried out using the Benjamini–Hochberg method. Cut-offs for log2 fold changes and *P*-values are given in Supplemental Fig. S1. The mass spectrometry proteomics data have been deposited to the ProteomeXchange Consortium via the PRIDE (Perez-Riverol et al. 2019) partner repository with the dataset identifier PXD038009 and 10.6019/PXD038009 .

### Measurement of redox activity

Expression and purification of recombinant proteins, including mutated versions, were as described earlier (Hochmal et al. 2016; Marchetti et al. 2022). To determine NTRC redox activity, 400 nM NTRC were reduced by 200 µM NADPH at a free calcium concentration of 0 to 2.5 µM for 10 min at RT in 30 mM MOPS, 100 mM KCl, pH 7.2 in the presence of different concentrations of CRX WT, its active site mutant (C238S, C242S named C1,2S here), TRX f and TRXR, the in vitro electron donor of CRX, as indicated in the corresponding figures. After addition of 200 mM DTNB, the formation of TNB^−^ was measured at 412 nm (Ultrospec 3000, Amersham Biosciences). The reduction rate of DNTB was determined over a time course of 0 to 80 s after addition of DTNB. The different activities were normalized to the highest activity measured for each batch of purified NTRC. For CRX, 1 µM CRX were reduced as described for NTRC in the presence of 200 nM TRXR and different concentration of NTRC-C136S. Reaction and reduction rates were carried out and calculated as described.

### PRX1 interaction assay

The interaction between NTRC, CRX, and PRX1 was measured in vitro via a photometrical assay (Nelson and Parsonage 2011). The 5 µM recombinant NTRC was reduced at RT by incubation with 120 µM NADPH and 80 µM H_2_O_2_ in 30 mM MOPS, 100 mM KCl, pH 7.2 at a free calcium concentration of 2.5 µM in the presence of different concentrations of CRX WT and TRXR. The NADPH consumption was measured at 340 nm until a steady decrease in absorption was detected. The reaction was then started by the addition of 1 µM recombinant PRX1, and recording of the absorption at 340 nm was continued. The rate of NADPH oxidation was calculated from the first 60 s after the addition of PRX1.

### NTRC and CRX absolute quantification by mass spectrometry

The LC-MS/MS system for sample analysis was identical to the one used for the whole proteome analysis. Recombinant CRX and NTRC (vectors described in Hochmal et al. (2016) and Marchetti et al. (2022) were expressed by *E. coli* cultures grown in ^15^N-labeling medium following (Nikolova et al. 2018). Protein purification was done as described (Hochmal et al. 2016; Marchetti et al. 2022). Cell lysates obtained from four independent unlabeled (^14^N) biological replicates were mixed at equal ratios. Mixed samples were then divided into three aliquots, each containing 50 µg protein (^14^N) plus a known amount (25, 125, or 500 fmol) of the two ^15^N-labeled proteins. Samples were then subjected to tryptic digestion following the FASP protocol described above. Peptide aliquots corresponding to 5 µg of total protein (^14^N) were desalted, dried by vacuum centrifugation and resuspended in 5 µl of loading buffer (see above). A sample volume of 1 µl was injected, corresponding to 1 µg of ^14^N-labeled peptides plus 0.5, 2.5, or 10 fmol of ^15^N-labeled CRX and NTRC peptides. The mass spectrometer was operated in parallel reaction monitoring (PRM) mode, targeting the light and heavy versions of five distinct peptides per protein. MS raw files were loaded into Skyline (MacLean et al. 2010) for peak extraction and integration of ^14^N and ^15^N peptide signals. From the peptide peak areas of four ^15^N peptides for each protein, calibration curves were generated, which were then used to calculate the absolute amounts of each ^14^N peptide. The protein amount corresponded to the mean value of the peak areas of the respective four ^14^N peptides of CRX and NTRC. Standard deviations relate to the results obtained from the calculations based on the four different peptides used.

### Non-reducing SDS gels

To visualize the PRX1 redox state, non-reducing SDS gels were run using normal Laemmli-loading buffer but omitting DTT and β-mercaptoethanol. One microgram of recombinant PRX1 (vector and purification described in (Charoenwattanasatien et al. 2018)) was reduced or oxidized with 1 mM DTT or H_2_O_2_ for 1 h at RT. The reducing/oxidizing agent was washed out over a G25 column (GE Healthcare) following the manufacturer's instructions using 30 mM MOPS, 100 mM KCl, pH 7.2 as buffering agent. Equal amounts of CRX, NTRC, or mutated versions were mixed with reduced or oxidized PRX1, as indicated in the figure. After incubation for 1 h at RT, non-red loading dye was added, and samples were heated to 70°C for 20 min before loading onto SDS-PAGE gels.

### NEM labeling

Recombinant PRX1 was blocked in its respective redox state by TCA precipitation at a final concentration of 10% (w/v). After incubation at −20°C for at least 2 h and centrifugation (16 000×g, 15 min, 4°C), precipitates were washed with 1% (w/v) TCA in acetone, centrifuged again and then washed with 100% acetone. Washing steps were performed on ice. After another centrifugation step, acetone was removed, and the pellet was let dry for approximately 20 min at RT. Pellets were resuspended in 50 mM N-ethyl-maleimide (NEM) in 8 M Urea, 2% (w/v) SDS, 100 mM Tris/HCl, pH 7.5, 10 mM EDTA for 2 h at RT to block reduced cysteines. After another TCA precipitation step (10% (w/v) final concentration, 2 h −20°C followed by centrifugation as described above), pellets were washed with 100% acetone twice and resuspended in non-reducing loading dye for SDS-PAGE.

### LC-MS/MS analysis of NEM-labeled PRX1

Gel bands containing PRX1 were cut out of the gel and subjected to tryptic in-gel digestion according to established protocols, including the reduction and alkylation of non-NEM-labeled cysteines (Shevchenko et al. 2006). After desalting resulting peptides using C18 STAGE tips (Rappsilber et al. 2007), LC-MS/MS analysis was carried out on the system described above. Samples were loaded on the trap column for 5 min at a flow rate of 10 µl/min using 2.5% loading buffer. Then, peptides were separated with the following gradient: 2.5% to 40% B over 55 min, 40% to 99% B over 5 min, 99% B for 20 min. MS data acquisition settings were as described for the whole proteome analysis with the following changes: MS/MS maximum injection time was 55 ms, and the intensity threshold was 1e4. Dynamic exclusion was set to “auto,” assuming a chromatographic peak width (FWHM) of 10 s.

Spectra files were searched in Proteome Discoverer 2.4 against the polypeptide sequence of recombinant PRX1, common contaminants (cRAP, www.thegpm.org/crap/), and the *E. coli* proteome (Uniprot ID UP000000625) using the MSFragger, MS Amanda, and Sequest HT nodes. Default search parameters for data acquisition in the Orbitrap were used with the following exceptions, all regarding the modification of cysteine residues: NEM labeling, carbamidomethylation, oxidation, dioxidation, and trioxidation were chosen as variable modifications. Peptide precursor intensities were determined via the Minora feature detector node in Proteome Discoverer. Ion traces of the NEM-labeled and the carbamidomethylated versions of the PRX1 peptide VLQAIQYVQSNPDEVCPAGWKPGDK were exported directly from the software. The double peak observed for the NEM derivative is most likely due to the presence of two diastereoisomers resulting from the introduction of a new chiral center by the labeling (Jemal and Hawthorne 1994; Srinivas and Mamidi 2003).

### Assessment of NTRC cysteines redox state

Recombinant NTRC was incubated alone or with 10 times more recombinant CRX or TRX f in 30 mM MOPS, 100 mM KCl, 2 mM NADPH at a free Ca^2+^ concentration of 0 µM for 10 min. 50 µg of protein mixtures were labeled with 40 mM chloroacetic acid (CAA) at room temperature for 1 h. After removal of residual CAA by washing four times with 50 mM ammonium bicarbonate (ABC) in centrifugal filter devices (Amicon Ultra 30 K, 0.5 ml, Merck), denaturation and reduction of non-labeled cysteines were achieved by adding 10 mM TCEP in 8 mM Urea, 100 mM Tris/HCl, pH 8.5 and incubation for 1 h at 25°C. The same conditions were applied for subsequent cysteine labeling with 4-vinylpyridine (VP, 50 mM) labeling. After four washes with 50 mM ABC to remove excess VP, samples were tryptically digested and prepared for MS analysis following the FASP protocol as described above.

### Quantification of differentially labeled cysteine-containing NTRC peptides by parallel reaction monitoring (PRM)

Twenty-five nanograms of labeled and digested NTRC were analyzed on the same LC-MS/MS system using the same buffers and loading conditions described above. Peptide separation was carried out on a PepsSep 15 reversed-phase column (75 µm × 15 cm, 1.9 µm particle size, 100 Å pore size; Bruker). The gradient applied was as follows: 5% to 40% B over 30 min, 40% to 99% over 2 min, 99% B over 5 min. Two rounds of sample analysis were performed. In the first round, samples were analyzed in conventional DDA mode (see above). A spectral library was constructed using BiblioSpec in Skyline 22.2.0.351 (Frewen and MacCoss 2007; MacLean et al. 2010) by making use of peptide identifications obtained by searching spectra files with MSFragger (Kong et al. 2017) against a concatenated database consisting of the protein sequences of recombinant NTRC and common contaminants (Frankenfield et al. 2022), as well as the *E. coli* proteome (Uniprot UP000000625). Carbamidomethylation, vinylpyridination, and trioxidation of cysteine, oxidation of methionine, and acetylation of protein N-termini were allowed as variable modifications.

Using the spectral library, an inclusion list of NTRC peptides was generated, and samples were analyzed again, this time in PRM-mode (parallel reaction monitoring) for targeted quantification (120 ms maximum injection time, AGC target 1e5, isolation window 2 *m*/*z*, isolation offset 0.5 *m*/*z*, resolution 35,000). Raw files acquired in PRM mode were loaded in Skyline for peptide identification and peak integration. By default, a minimum of six fragment ions per peptide were used for quantification. For positive peptide identification, a peptide dotp of >0.9 was required. If minimum requirements for identification and quantification were satisfied in at least one replicate, the presence of at least three fragment ions with a maximum retention time shift of 30 s and a dotp of 0.7 were considered sufficient for quantification. Data normalization was performed using the “global standards” method, i.e. the ratio of the total peak area of a modified peptide and the sum of the total peak areas of two NTRC peptides lacking cysteine (ANLKPVVFEGFR, LVAGQVELDEAGYVK) was calculated. Origin 2023 Pro was used for the statistical analysis of peptide abundance data (one-way ANOVA followed by Tukey's honest different test).

### Membrane inlet mass spectrometry

To compare the physiology of the mutated CRX strains (E1 and A5) to their background wild-type strain (CC125) as well as IM*_crx_* and IM-R2, cells were grown in TP medium and kept at 24.5°C under a continuous irradiance of 30 µmol photons·m^−2^·s^−1^ (referred to as LL). When stated, cells were exposed to 500 µmol photons·m^−2^·s^−1^ for 12 h prior to measurements (referred to as HL). The cells were then harvested, centrifuged at 3300×g for 2 min and resuspended to a final concentration of 15 µg/ml chlorophyll in TP medium, supplemented with 50 mM HEPES and 2 mM Na_2_CO_3_, at pH 7.2. Photosynthetic activity was assessed by a combined home-built membrane inlet mass spectrometer (MIMS) and a dual pulse amplitude modulated fluorometer. The measured masses were 32, 36, 40, and 44, corresponding to ^16^O_2_, ^18^O_2_, Ar, and CO_2_, respectively. The cells were then maintained in a short dark regime, during which their quantum efficiency (F_v_/F_m_) was determined by exposing them to a saturating pulse (10 mmol photons·m^−2^·s^−1^, 300 ms). During measurements, the cells were exposed to HL (500 µmol photons·m^−2^·s^−1^, mixed red/blue light) for 3 min, followed by darkness for 3 min. This scheme was repeated twice in order to decrease noise and verify that no further changes were generated by the short light exposure time. Net O_2_ evolution was assessed by tracking ^16^O_2_ concentration during light exposure. Gross O_2_ evolution was assessed by subtracting ^18^O_2_ uptake in light relative to dark exposures from net O_2_ evolution, as was previously demonstrated by Kedem et al. (2021). CO_2_ fixation was assessed by subtracting the rate of CO_2_ uptake during light exposure from its evolution rate under darkness.

### Accession numbers

Sequence data from this article can be found in the ProteinDataBase (PDB) under accession numbers 5E37 (CRX), 7P9E (NTRC), and 6J13 (PRX1).

## Supplementary Material

kiad426_Supplementary_Data

## Supplemental data

The following materials are available in the online version of this article.

**Supplemental Figure S1.** Volcano plots of quantitative (LFQ) proteomics data.

## References

[kiad426-B1] Bassham JA , Benson AA, Calvin M. The path of carbon in photosynthesis. J Biol Chem. 1950:185:781–787. 14774424

[kiad426-B2] Bec Kova M , Yu J, Krynicka V, Kozlo A, Shao S, Konik P, Komenda J, Murray JW, Nixon PJ. Structure of Psb29/Thf1 and its association with the FtsH protease complex involved in photosystem II repair in cyanobacteria. Philos Trans R Soc Lond B Biol Sci. 2017:372(1730):20160394. 10.1098/rstb.2016.0394 PMC5566888 28808107

[kiad426-B3] Carrillo LR , Froehlich JE, Cruz JA, Savage LJ, Kramer DM. Multi-level regulation of the chloroplast ATP synthase: the chloroplast NADPH thioredoxin reductase C (NTRC) is required for redox modulation specifically under low irradiance. Plant J. 2016:87(6):654–663. 10.1111/tpj.13226 27233821

[kiad426-B4] Chae HB , Moon JC, Shin MR, Chi YH, Jung YJ, Lee SY, Nawkar GM, Jung HS, Hyun JK, Kim WY, et al Thioredoxin reductase type C (NTRC) orchestrates enhanced thermotolerance to Arabidopsis by its redox-dependent holdase chaperone function. Mol Plant. 2013:6(2):323–336. 10.1093/mp/sss105 23024205

[kiad426-B5] Charoenwattanasatien R , Tanaka H, Zinzius K, Hochmal AK, Mutoh R, Yamamoto D, Hippler M, Kurisu G. X-ray crystallographic and high-speed AFM studies of peroxiredoxin 1 from *Chlamydomonas reinhardtii*. Acta Crystallogr F Struct Biol Commun. 2018:74(2):86–91. 10.1107/S2053230X17018507 29400317 PMC5947678

[kiad426-B6] Charoenwattanasatien R , Zinzius K, Scholz M, Wicke S, Tanaka H, Brandenburg JS, Marchetti GM, Ikegami T, Matsumoto T, Oda T, et al Calcium sensing via EF-hand 4 enables thioredoxin activity in the sensor-responder protein calredoxin in the green alga *Chlamydomonas reinhardtii*. J Biol Chem. 2020:295(1):170–180. 10.1074/jbc.RA119.008735 31776187 PMC6952598

[kiad426-B7] da Veiga Leprevost F , Haynes SE, Avtonomov DM, Chang H-Y, Shanmugam AK, Mellacheruvu D, Kong AT, Nesvizhskii AI. Philosopher: a versatile toolkit for shotgun proteomics data analysis. Nat Methods. 2020:17(9):869–870. 10.1038/s41592-020-0912-y 32669682 PMC7509848

[kiad426-B8] Dietz K-J . Plant peroxiredoxins. Annu Rev Plant Biol. 2003:54:93–107. 10.1146/annurev.arplant.54.031902.134934. 14502986

[kiad426-B10] Dietz KJ . Peroxiredoxins in plants and cyanobacteria. Antioxid Redox Signal. 2011:15:1129–1159. 21194355 10.1089/ars.2010.3657 PMC3135184

[kiad426-B9] Dietz KJ , Jacob S, Oelze ML, Laxa M, Tognetti V, de Miranda SM, Baier M, Finkemeier I. The function of peroxiredoxins in plant organelle redox metabolism. J Exp Bot. 2006:57(8):1697–1709. 10.1093/jxb/erj160 16606633

[kiad426-B11] Esteban-Fernández D , Scheler C, Linscheid MW. Absolute protein quantification by LC-ICP-MS using MeCAT peptide labeling. Anal Bioanal Chem. 2011:401(2):657–666. 10.1007/s00216-011-5104-2 21626189

[kiad426-B12] Flowers JM , Hazzouri KM, Pham GM, Rosas U, Bahmani T, Khraiwesh B, Nelson DR, Jijakli K, Abdrabu R, Harris EH, et al Whole-genome resequencing reveals extensive natural variation in the model green alga *Chlamydomonas reinhardtii*. Plant Cell. 2015:27(9):2353–2369. 10.1105/tpc.15.00492 26392080 PMC4815094

[kiad426-B13] Foyer CH . Reactive oxygen species, oxidative signaling and the regulation of photosynthesis. Environ Exp Bot. 2018:154:134–142. 30283160 10.1016/j.envexpbot.2018.05.003 PMC6105748

[kiad426-B14] Frankenfield AM , Ni J, Ahmed M, Hao L. Protein contaminants matter: building universal protein contaminant libraries for DDA and DIA proteomics. J Proteome Res. 2022:21(9):2104–2113. 10.1021/acs.jproteome.2c00145 35793413 PMC10040255

[kiad426-B15] Frewen B , MacCoss MJ. Using BiblioSpec for creating and searching tandem MS peptide libraries. Curr Protoc Bioinformatics. 2007:Chapter 13:13.17.1–13.17.12. 10.1002/0471250953.bi1307s20 18428681

[kiad426-B16] Glaesener AG , Merchant SS, Blaby-Haas CE. Iron economy in *Chlamydomonas reinhardtii*. Front Plant Sci. 2013:4:337. 10.3389/fpls.2013.00337 24032036 PMC3759009

[kiad426-B17] Greiner A , Kelterborn S, Evers H, Kreimer G, Sizova I, Hegemann P. Targeting of photoreceptor genes in *Chlamydomonas reinhardtii* via zinc-finger nucleases and CRISPR/cas9. Plant Cell. 2017:29(10):2498–2518. 10.1105/tpc.17.00659 28978758 PMC5774583

[kiad426-B18] Guinea Diaz M , Nikkanen L, Himanen K, Toivola J, Rintamaki E. Two chloroplast thioredoxin systems differentially modulate photosynthesis in Arabidopsis depending on light intensity and leaf age. Plant J. 2020:104(3):718–734. 10.1111/tpj.14959 32772439 PMC7693050

[kiad426-B19] Hochmal AK , Schulze S, Trompelt K, Hippler M. Calcium-dependent regulation of photosynthesis. Biochim Biophys Acta. 2015:1847(9):993–1003. 10.1016/j.bbabio.2015.02.010 25687895

[kiad426-B20] Hochmal AK , Zinzius K, Charoenwattanasatien R, Gabelein P, Mutoh R, Tanaka H, Schulze S, Liu G, Scholz M, Nordhues A, et al Calredoxin represents a novel type of calcium-dependent sensor-responder connected to redox regulation in the chloroplast. Nat Commun. 2016:7(1):11847. 10.1038/ncomms11847 27297041 PMC4911631

[kiad426-B21] Hou LY , Ehrlich M, Thormählen I, Lehmann M, Krahnert I, Obata T, Cejudo FJ, Fernie AR, Geigenberger P. NTRC plays a crucial role in starch metabolism, redox balance, and tomato fruit growth. Plant Physiol. 2019:181(3):976–992. 10.1104/pp.19.00911 31527089 PMC6836810

[kiad426-B22] Hulstaert N , Shofstahl J, Sachsenberg T, Walzer M, Barsnes H, Martens L, Perez-Riverol Y. Thermorawfileparser: modular, scalable, and cross-platform RAW file conversion. J Proteome Res. 2020:19(1):537–542. 10.1021/acs.jproteome.9b00328 31755270 PMC7116465

[kiad426-B23] Jemal M , Hawthorne D. High performance liquid chromatography/ionspray mass spectrometry of N-ethylmaleimide and acrylic acid ester derivatives for bioanalysis of thiol compounds. Rapid Commun Mass Spectrom. 1994:8(10):854–857. 10.1002/rcm.1290081012

[kiad426-B24] Johnson CH , Knight MR, Kondo T, Masson P, Sedbrook J, Haley A, Trewavas A. Circadian oscillations of cytosolic and chloroplastic free calcium in plants. Science 1995:269:1863–1865. 7569925 10.1126/science.7569925

[kiad426-B25] Kedem I , Treves H, Noble G, Hagemann M, Murik O, Raanan H, Oren N, Giordano M, Kaplan A. Keep your friends close and your competitors closer: novel interspecies interaction in desert biological sand crusts. Phycologia. 2021:60(5):419–426. 10.1080/00318884.2020.1843349

[kiad426-B26] Kelterborn S , Boehning F, Sizova I, Baidukova O, Evers H, Hegemann P. Gene editing in green alga *Chlamydomonas reinhardtii* via CRISPR-cas9 ribonucleoproteins. In: Zurbriggen MD, editor. Plant synthetic biology: methods and protocols. New York (NY): Springer US; 2022. p. 45–65. 10.1007/978-1-0716-1791-5_3 35188655

[kiad426-B27] Kong AT , Leprevost FV, Avtonomov DM, Mellacheruvu D, Nesvizhskii AI. MSFragger: ultrafast and comprehensive peptide identification in mass spectrometry-based proteomics. Nat Methods. 2017:14(5):513–520. 10.1038/nmeth.4256 28394336 PMC5409104

[kiad426-B28] Kudla J , Becker D, Grill E, Hedrich R, Hippler M, Kummer U, Parniske M, Romeis T, Schumacher K. Advances and current challenges in calcium signaling. New Phytol. 2018:218(2):414–431. 10.1111/nph.14966 29332310

[kiad426-B29] Lepisto A , Pakula E, Toivola J, Krieger-Liszkay A, Vignols F, Rintamaki E. Deletion of chloroplast NADPH-dependent thioredoxin reductase results in inability to regulate starch synthesis and causes stunted growth under short-day photoperiods. J Exp Bot. 2013:64(12):3843–3854. 10.1093/jxb/ert216 23881397 PMC3745738

[kiad426-B30] MacLean B , Tomazela DM, Shulman N, Chambers M, Finney GL, Frewen B, Kern R, Tabb DL, Liebler DC, MacCoss MJ. Skyline: an open source document editor for creating and analyzing targeted proteomics experiments. Bioinformatics. 2010:26(7):966–968. 10.1093/bioinformatics/btq054 20147306 PMC2844992

[kiad426-B31] Marchetti GM , Fusser F, Singh RK, Brummel M, Koch O, Kummel D, Hippler M. Structural analysis revealed a novel conformation of the NTRC reductase domain from *Chlamydomonas reinhardtii*. J Struct Biol. 2022:214(1):107829. 10.1016/j.jsb.2021.107829 34974142

[kiad426-B32] Mitchell P . Coupling of phosphorylation to electron and hydrogen transfer by a chemi-osmotic type of mechanism. Nature 1961:191:144–148. 13771349 10.1038/191144a0

[kiad426-B33] Naranjo B , Mignee C, Krieger-Liszkay A, Hornero-Mendez D, Gallardo-Guerrero L, Cejudo FJ, Lindahl M. The chloroplast NADPH thioredoxin reductase C, NTRC, controls non-photochemical quenching of light energy and photosynthetic electron transport in Arabidopsis. Plant Cell Environ. 2016:39(4):804–822. 10.1111/pce.12652 26476233

[kiad426-B34] Nelson KJ , Parsonage D. Measurement of peroxiredoxin activity. Curr Protoc Toxicol. 2011:Chapter 7:Unit7.10. 10.1002/0471140856.tx0710s49 PMC3703631 21818753

[kiad426-B35] Nikkanen L , Toivola J, Rintamäki E. Crosstalk between chloroplast thioredoxin systems in regulation of photosynthesis. Plant Cell Environ. 2016:39(8):1691–1705. 10.1111/pce.12718 26831830

[kiad426-B36] Nikolova D , Heilmann C, Hawat S, Gäbelein P, Hippler M. Absolute quantification of selected photosynthetic electron transfer proteins in *Chlamydomonas reinhardtii* in the presence and absence of oxygen. Photosynth Res. 2018:137(2):281–293. 10.1007/s11120-018-0502-3 29594952

[kiad426-B37] Noctor G , Reichheld JP, Foyer CH. ROS-related redox regulation and signaling in plants. Semin Cell Dev Biol. 2018:80:3–12. 28733165 10.1016/j.semcdb.2017.07.013

[kiad426-B38] Ojeda V , Pérez-Ruiz JM, Cejudo FJ. 2-Cys peroxiredoxins participate in the oxidation of chloroplast enzymes in the dark. Mol Plant. 2018:11(11):1377–1388. 10.1016/j.molp.2018.09.005 30292682

[kiad426-B39] Pascual MB , Mata-Cabana A, Florencio FJ, Lindahl M, Cejudo FJ. A comparative analysis of the NADPH thioredoxin reductase C-2-Cys peroxiredoxin system from plants and cyanobacteria. Plant Physiol. 2011:155(4):1806–1816. 10.1104/pp.110.171082 21335525 PMC3091103

[kiad426-B40] Perez-Riverol Y , Csordas A, Bai JW, Bernal-Llinares M, Hewapathirana S, Kundu DJ, Inuganti A, Griss J, Mayer G, Eisenacher M, et al The PRIDE database and related tools and resources in 2019: improving support for quantification data. Nucleic Acids Res. 2019:47(D1):D442–D450. 10.1093/nar/gky1106 30395289 PMC6323896

[kiad426-B41] Pérez-Ruiz JM , Cejudo FJ. A proposed reaction mechanism for rice NADPH thioredoxin reductase C, an enzyme with protein disulfide reductase activity. FEBS Lett. 2009:583(9):1399–1402. 10.1016/j.febslet.2009.03.067 19345687

[kiad426-B42] Pérez-Ruiz JM , Naranjo B, Ojeda V, Guinea M, Cejudo FJ. NTRC-dependent redox balance of 2-Cys peroxiredoxins is needed for optimal function of the photosynthetic apparatus. Proc Natl Acad Sci USA. 2017:114(45):12069–12074. 10.1073/pnas.1706003114 29078290 PMC5692536

[kiad426-B43] Pérez-Ruiz JM , Spinola MC, Kirchsteiger K, Moreno J, Sahrawy M, Cejudo FJ. Rice NTRC is a high-efficiency redox system for chloroplast protection against oxidative damage. Plant Cell. 2006:18(9):2356–2368. 10.1105/tpc.106.041541 16891402 PMC1560923

[kiad426-B44] Pirayesh N , Giridhar M, Ben Khedher A, Vothknecht UC, Chigri F. Organellar calcium signaling in plants: an update. Biochim Biophys Acta Mol Cell Res. 2021:1868(4):118948. 10.1016/j.bbamcr.2021.118948 33421535

[kiad426-B45] Poole LB , Karplus PA, Claiborne A. Protein sulfenic acids in redox signaling. Annu Rev Pharmacol Toxicol. 2004:44(1):325–347. 10.1146/annurev.pharmtox.44.101802.121735 14744249

[kiad426-B46] Pulido P , Spinola MC, Kirchsteiger K, Guinea M, Pascual MB, Sahrawy M, Sandalio LM, Dietz KJ, Gonzalez M, Cejudo FJ. Functional analysis of the pathways for 2-Cys peroxiredoxin reduction in *Arabidopsis thaliana* chloroplasts. J Exp Bot. 2010:61(14):4043–4054. 10.1093/jxb/erq218 20616155 PMC2935875

[kiad426-B47] Queitsch C , Sangster TA, Lindquist S. Hsp90 as a capacitor of phenotypic variation. Nature. 2002:417(6889):618–624. 10.1038/nature749 12050657

[kiad426-B48] Rappsilber J , Ishihama Y, Mann M. Stop and go extraction tips for matrix-assisted laser desorption/ionization, nanoelectrospray, and LC/MS sample pretreatment in proteomics. Anal Chem. 2003:75(3):663–670. 10.1021/ac026117i 12585499

[kiad426-B49] Rappsilber J , Mann M, Ishihama Y. Protocol for micro-purification, enrichment, pre-fractionation and storage of peptides for proteomics using StageTips. Nat Protoc. 2007:2(8):1896–1906. 10.1038/nprot.2007.261 17703201

[kiad426-B50] Richter AS , Peter E, Rothbart M, Schlicke H, Toivola J, Rintamki E, Grimm B. Posttranslational influence of NADPH-dependent thioredoxin reductase C on enzymes in tetrapyrrole synthesis. Plant Physiol. 2013:162(1):63–73. 10.1104/pp.113.217141 23569108 PMC3641230

[kiad426-B51] Ritchie ME , Phipson B, Wu D, Hu Y, Law CW, Shi W, Smyth GK. Limma powers differential expression analyses for RNA-sequencing and microarray studies. Nucleic Acids Res. 2015:43(7):e47. 10.1093/nar/gkv007 25605792 PMC4402510

[kiad426-B52] Sakamoto W , Tamura T, Hanba-Tomita Y, Murata M. The VAR1 locus of Arabidopsis encodes a chloroplastic FtsH and is responsible for leaf variegation in the mutant alleles. Genes Cells. 2002:7(8):769–780. 10.1046/j.1365-2443.2002.00558.x 12167156

[kiad426-B53] Serrato AJ , Perez-Ruiz JM, Spinola MC, Cejudo FJ. A novel NADPH thioredoxin reductase, localized in the chloroplast, which deficiency causes hypersensitivity to abiotic stress in *Arabidopsis thaliana*. J Biol Chem. 2004:279(42):43821–43827. 10.1074/jbc.M404696200 15292215

[kiad426-B54] Shah AD , Goode RJA, Huang C, Powell DR, Schittenhelm RB. LFQ-Analyst: an easy-to-use interactive web platform to analyze and visualize label-free proteomics data preprocessed with MaxQuant. J Proteome Res. 2020:19(1):204–211. 10.1021/acs.jproteome.9b00496 31657565

[kiad426-B55] Shevchenko A , Tomas H, Havlis J, Olsen JV, Mann M. In-gel digestion for mass spectrometric characterization of proteins and proteomes. Nat Protoc. 2006:1(6):2856–2860. 10.1038/nprot.2006.468 17406544

[kiad426-B56] Smyth DG , Blumenfeld OO, Konigsberg W. Reactions of N-ethylmaleimide with peptides and amino acids. Biochem J. 1964:91(3):589–595. 10.1042/bj0910589 5840721 PMC1202996

[kiad426-B57] Sobotta MC , Liou W, Stöcker S, Talwar D, Oehler M, Ruppert T, Scharf AN, Dick TP. Peroxiredoxin-2 and STAT3 form a redox relay for H2O2 signaling. Nat Chem Biol. 2015:11(1):64–70. 10.1038/nchembio.1695 25402766

[kiad426-B58] Srinivas NR , Mamidi RNVS. Bioanalytical considerations for compounds containing free sulfhydryl groups. Biomed Chromatogr. 2003:17(5):285–291. 10.1002/bmc.256 12884392

[kiad426-B59] Thormahlen I , Meitzel T, Groysman J, Ochsner AB, von Roepenack-Lahaye E, Naranjo B, Cejudo FJ, Geigenberger P. Thioredoxin f1 and NADPH-dependent thioredoxin reductase C have overlapping functions in regulating photosynthetic metabolism and plant growth in response to varying light conditions. Plant Physiol. 2015:169(3):1766–1786. 10.1104/pp.15.01122 26338951 PMC4634086

[kiad426-B60] Thormahlen I , Zupok A, Rescher J, Leger J, Weissenberger S, Groysman J, Orwat A, Chatel-Innocenti G, Issakidis-Bourguet E, Armbruster U, et al Thioredoxins play a crucial role in dynamic acclimation of photosynthesis in fluctuating light. Mol Plant. 2017:10(1):168–182. 10.1016/j.molp.2016.11.012 27940305

[kiad426-B61] Tyanova S , Temu T, Sinitcyn P, Carlson A, Hein MY, Geiger T, Mann M, Cox J. The Perseus computational platform for comprehensive analysis of (prote)omics data. Nat Methods. 2016:13(9):731–740. 10.1038/nmeth.3901 27348712

[kiad426-B62] van den Hooven HW , van den Burg HA, Vossen P, Boeren S, de Wit PJ, Vervoort J. Disulfide bond structure of the AVR9 elicitor of the fungal tomato pathogen *Cladosporium fulvum*: evidence for a cystine knot. Biochemistry. 2001:40(12):3458–3466. 10.1021/bi0023089 11297411

[kiad426-B63] Whatley FR , Arnon DI, Tagawa K. Separation of light and dark reactions in electron transfer during photosynthesis. Proc Natl Acad Sci USA. 1963:49:266–270. 14000214 10.1073/pnas.49.2.266 PMC299795

[kiad426-B64] Wiśniewski JR , Zougman A, Nagaraj N, Mann M. Universal sample preparation method for proteome analysis. Nat Methods. 2009:6(5):359–362. 10.1038/nmeth.1322 19377485

[kiad426-B65] Yoshida K , Hisabori T. Two distinct redox cascades cooperatively regulate chloroplast functions and sustain plant viability. Proc Natl Acad Sci USA. 2016:113(27):E3967–E3976. 10.1073/pnas.1604101113 27335455 PMC4941451

[kiad426-B66] Yoshida K , Hisabori T. Distinct electron transfer from ferredoxin-thioredoxin reductase to multiple thioredoxin isoforms in chloroplasts. Biochem J. 2017:474(8):1347–1360. 10.1042/BCJ20161089 28246333

[kiad426-B67] Yu F , Haynes SE, Teo GC, Avtonomov DM, Polasky DA, Nesvizhskii AI. Fast quantitative analysis of timsTOF PASEF data with MSFragger and IonQuant. Mol Cell Proteomics 2020:19(9):1575–1585 10.1074/mcp.TIR120.002048 32616513 PMC7996969

[kiad426-B68] Zhang L , Wei Q, Wu W, Cheng Y, Hu G, Hu F, Sun Y, Zhu Y, Sakamoto W, Huang J. Activation of the heterotrimeric G protein alpha-subunit GPA1 suppresses the ftsh-mediated inhibition of chloroplast development in Arabidopsis. Plant J. 2009:58(6):1041–1053. 10.1111/j.1365-313X.2009.03843.x 19228339

