# Supplementary material for: Calredoxin regulates the chloroplast NADPH-dependent thioredoxin reductase in Chlamydomonas reinhardtii
Source: Plant Physiol. 2023 Jul 20;193(3):2122–40. doi: 10.1093/plphys/kiad426 (PMC10602609; doi:10.1093/plphys/kiad426)
Supplement: kiad426_Supplementary_Data [file kiad426_supplementary_data.zip › kiad426_Supplementary_Data.pdf]

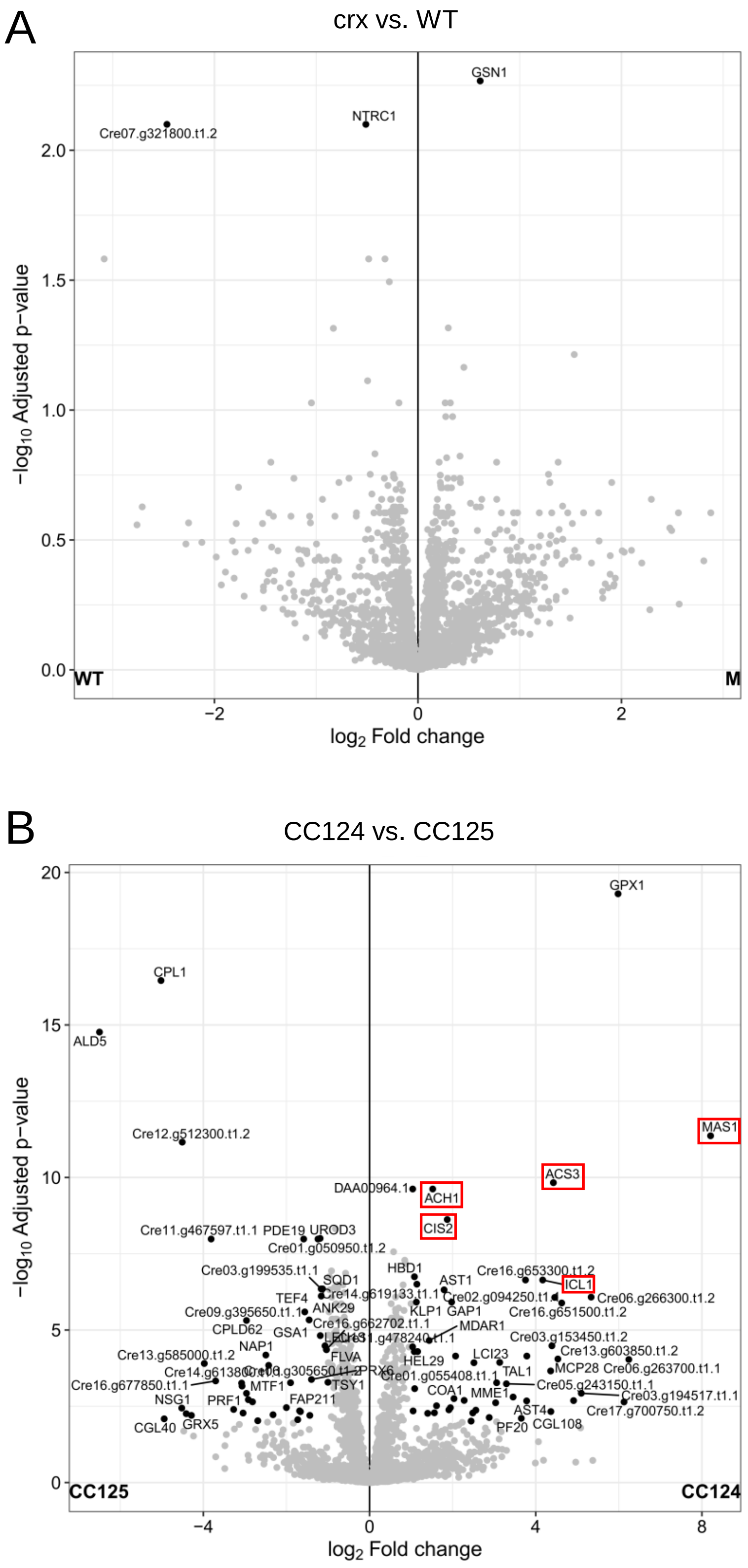

Supplemental Figure S1: Volcano plots of quantitative (LFQ) proteomics data. Log2 fold changes in protein abundances are plotted against Benjamini-Hochberg-adjusted p-values. Panel **(A)** displays combined data of all mutant strains versus all wildtypes. NTRC1 and FTSH1 (Cre01.g066552) are significantly less abundant in *crx* mutants (adj. p-value <0.01; $FC_{crx/WT}$ <-0.5, >0.5; n=12). **(B)** Volcano plot to identify differences in the proteomes of CC124 and CC125 wildtype strains. Proteins involved in acetate metabolism/glyoxylate cycle (red rectangles) are much more abundant in CC124. Differentially expressed proteins (adj. p-value <0.01; $FC_{CC124/CC125}$ <-1, >1; n=4) are highlighted in black.
